# A systematic characterization of microglia-like cell occurrence during retinal organoid differentiation

**DOI:** 10.1016/j.isci.2022.104580

**Published:** 2022-06-11

**Authors:** Katarina Bartalska, Verena Hübschmann, Medina Korkut-Demirbaş, Ryan John A. Cubero, Alessandro Venturino, Karl Rössler, Thomas Czech, Sandra Siegert

**Affiliations:** 1 Institute of Science and Technology Austria (ISTA), Am Campus 1, 3400 Klosterneuburg, Austria; 2 Medical University of Vienna, Department of Neurosurgery, Währinger Gürtel 18-20, 1090 Vienna, Austria

**Keywords:** Neuroscience, Sensory neuroscience, Cell biology

## Abstract

Cerebral organoids differentiated from human-induced pluripotent stem cells (hiPSC) provide a unique opportunity to investigate brain development. However, organoids usually lack microglia, brain-resident immune cells, which are present in the early embryonic brain and participate in neuronal circuit development. Here, we find IBA1^+^ microglia-like cells alongside retinal cups between week 3 and 4 in 2.5D culture with an unguided retinal organoid differentiation protocol. Microglia do not infiltrate the neuroectoderm and instead enrich within non-pigmented, 3D-cystic compartments that develop in parallel to the 3D-retinal organoids. When we guide the retinal organoid differentiation with low-dosed BMP4, we prevent cup development and enhance microglia and 3D-cysts formation. Mass spectrometry identifies these 3D-cysts to express mesenchymal and epithelial markers. We confirmed this microglia-preferred environment also within the unguided protocol, providing insight into microglial behavior and migration and offer a model to study how they enter and distribute within the human brain.

## Highlights

• Microglia-like cells occur alongside retinal cups in unguided hIPSC differentiation
• They populate non-pigmented 3D-cystic compartments
• The cystic compartment has a mesenchymal identity, which sequesters microglia
• Microglia-like cells adapt a BAM signature in cystic compartments

## Graphical abstract

**Figure:**
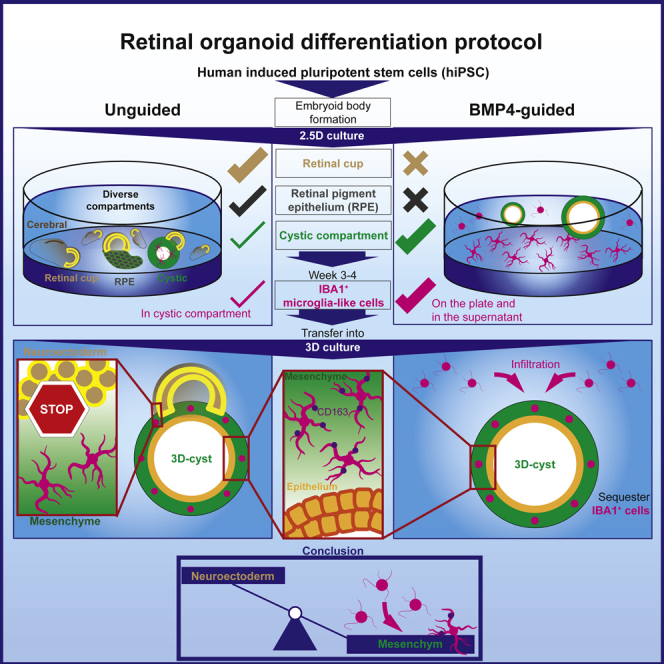

## Teaser

Microglia-like cells occur alongside human iPSC-derived retinal organoid differentiation and preferentially occupy the mesenchymal region.

## Introduction

The human brain consists of billions of neurons, glial and endothelial cells that self-organize during development into cellular networks, which perform distinct functions (Barresi, 2020). Microglia, the brain parenchymal immune cells, fine-tune neuronal circuits at the cellular and synaptic level (Cunningham et al., 2013; Guizzetti et al., 2014; Paolicelli and Gross, 2011; Schafer et al., 2012; Squarzoni et al., 2014). They derive from a primitive macrophage population, which develops within the yolk sac in both mice (Ginhoux et al., 2010) and in humans (Bian et al., 2020). Therefore, they represent a distinct macrophage population as they occur before the onset of hepatic and bone marrow hematopoiesis (Juul and Christensen, 2018; Menassa and Gomez-Nicola, 2018). Immunostaining of human embryonic brain tissue indicates that microglia enter the cerebral wall from the ventricular lumen and the leptomeninges at 4.5 gestation weeks and gradually colonize the cortex (Monier et al., 2007; Rezaie et al., 2005). The critical role of microglia in early human brain development has been further supported by hereditary mutations in macrophage-selective genes that cause numerous structural brain malformations in pediatric leukoencephalopathy (Oosterhof et al., 2019). A current bottleneck is the lack of accessible models that accurately recapitulate human microglia development, distribution, and action during circuit formation. So far, our knowledge is mostly limited to observations from postmortem fetal brain studies or nonhuman model systems like mice.

Human-induced pluripotent stem cells (hiPSC) have revolutionized the field of tissue engineering and allow exploring aspects of embryonic brain development (Bagley et al., 2017; Camp et al., 2015; Cowan et al., 2020; Lancaster et al., 2017). However, mesoderm-derived microglia are commonly lacking within cerebral organoids (Collin et al., 2019; Cowan et al., 2020; Kim et al., 2019; Lancaster and Knoblich, 2014). One likely explanation for this is that differentiation protocols often use supplements to direct hiPSC-formed embryoid bodies (EB) toward the neuroectodermal lineage to obtain cerebral organoids (Chambers et al., 2009; Pasca et al., 2015). To obtain human microglia-like cells, several groups have established guided protocols with BMP4 as a common nominator (Abud et al., 2017; Douvaras et al., 2017; Guttikonda et al., 2021; Haenseler et al., 2017; Pandya et al., 2017; Takata et al., 2017). Thus, several groups have assembled the hiPSC-derived microglia-like cells with separately derived cerebral organoids to analyze microglia function and interaction with neurons (Abud et al., 2017; Song et al., 2019; Xu et al., 2021), but this does not capture the natural progression of microglial appearance and distribution within brain tissue.

Unguided cerebral organoid differentiation provides an alternative strategy to capture various cell types. Here, EBs are cultured with minimal external interference and self-organize to a variety of cell lineage identities from fore-, mid-, and hindbrain (Qian et al., 2019). The hiPSC differentiation toward retinal organoids has been one of the first brain region-specific protocols (Eiraku et al., 2011; Nakano et al., 2012). This method reliably recapitulates the typical optical cup structure, expresses markers of well-defined cell types, and shows a light-sensitive response (Cowan et al., 2020; Zhong et al., 2014). In contrast, data are controversial regarding microglia occurrence in organoids. Although protocols report that microglia innately developed within cerebral organoids (Ormel et al., 2018) or single-cell RNA-sequencing identified a glial cluster expressing microglia-specific markers (Gabriel et al., 2021), other studies do not show or the provided data do not support their presence in retinal organoids (Collin et al., 2019; Cowan et al., 2020; Kim et al., 2019). On the other hand, microglia appear early in human embryonic retinal tissue at gestation week 5 as indicated by a microglial transcriptional signature (Hu et al., 2019; Mellough et al., 2019), and their localization within the human retinal layers by gestation week 10 (Diaz-Araya et al., 1995). To clarify whether microglia develop in unguided retinal organoid differentiation protocol (referred to from now on as unguided protocol), we implemented the protocol from (Zhong et al., 2014) and stained with the pan-macrophage marker IBA1/AIF1 (ionized calcium binding adaptor molecule one/allograft inflammatory factor 1), which identifies brain parenchymal- (microglia), blood-derived- (MΦ), and border-associated- (perivascular pvMΦ, leptomeningeal mMΦ, choroid plexus cpMΦ) macrophages (Imai et al., 1996; Ito et al., 2001; Kierdorf et al., 2019; Prinz and Priller, 2014). In 2.5D culture, we consistently found IBA1^+^-cells in parallel to developing retinal organoids by differentiation week 3 to 4. However, these IBA1^+^-cells rarely occupied the retinal or cerebral compartment and preferentially occurred in non-pigmented, cystic compartments that are commonly overlooked in organoid-focused studies.

Such cystic structures have also been mentioned in other microglia differentiation protocols (Haenseler et al., 2017; Muffat et al., 2016; Vaughan-Jackson et al., 2021). One common factor that is frequently used to enhance for microglia is BMP4. Therefore, we applied a low dose of BMP4 to the otherwise unchanged protocol to verify whether we can enrich for these 3D-cysts. We identified that these cysts highly express the mesenchymal and epithelial markers vimentin and E-Cadherin, respectively, and we confirmed a similar expression pattern in the cystic compartments in our unguided protocol. Finally, we found a strong overlap between IBA1 and CD163 expression, a marker for border-associated macrophages (BAMs) that reside either at perivascular structures, meninges or choroid plexus, all of mesenchymal nature (Lun et al., 2015; MacCord, 2012; Pill et al., 2015; Wimmer et al., 2019). The expression is turned on between week 5 and 6 in 2.5D culture.

In summary, our results confirm that IBA1^+^-cells exist in our unguided protocol, and we map their presence to cystic mesenchymal-like compartments, which codeveloped alongside 3D-retinal organoids. This work offers a model for exploring microglia integration during early development, and provides a foundation for future studies to dissect the molecular signaling mechanisms that attract microglia and foster their incorporation into cerebral organoids.

## Results

### IBA1^+^-microglia-like cells appear in the unguided protocol

**Figure 1:**
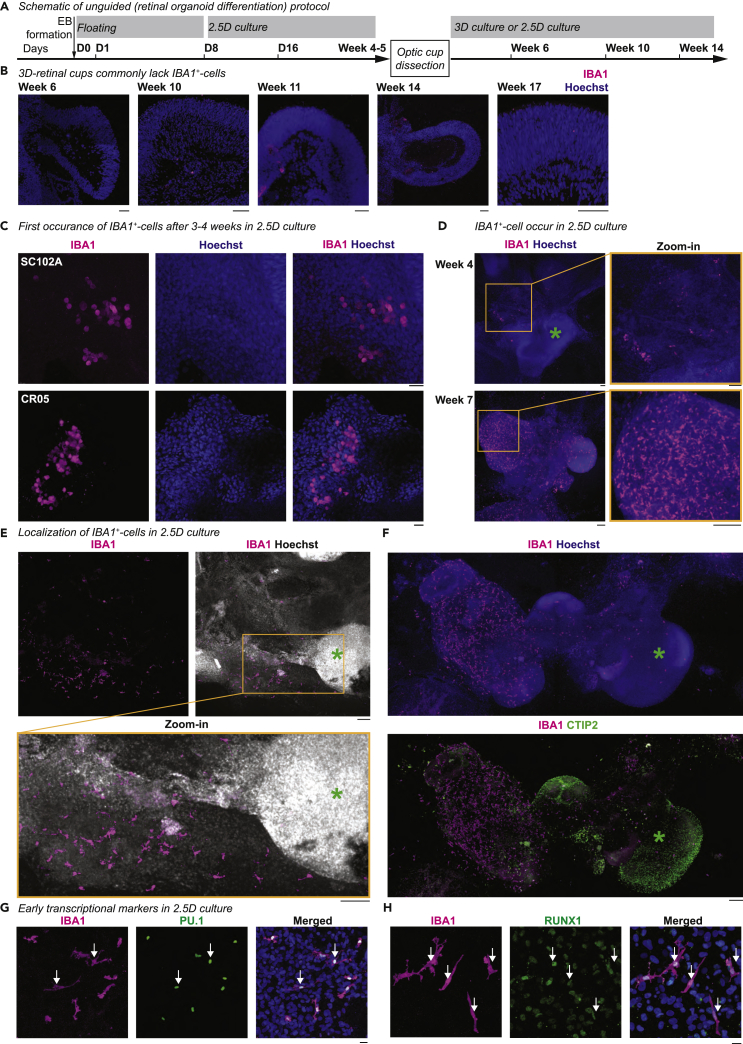
IBA1^+^-microglia-like cells occur during retinal organoid differentiation (A) Schematic of the unguided (retinal organoid differentiation) protocol (see also Figure S1A for detailed information). D, days after induced differentiation. EB, embryoid bodies. B-H, Immunostaining for IBA1 (ionized calcium-binding adapter molecule 1, magenta) with Hoechst to highlight nuclei (blue, except white in E). (B) Cryostat section of 3D-retinal organoids with focus on retinal cup at week 6, 10, 11, 14, and 17 for SC102A. Note: IBA1 staining occasionally occurred as a layered or dotted structure, which did not resolve in distinct cell morphologies. We excluded such staining patterns from further interpretations. Scale bar: 50 μm. (C–H) Staining in 2.5D culture. C, First occurrence of IBA1^+^-cells in SC102A (top) and CR05 (bottom) between week 3 and 4. Scale bar: 20 μm. D, SC102A at week 4 (top) and 7 (bottom). Scale bar: 100 μm. E, SC102A at week 9. Scale bar: 100 μm. F, SC102A at week 7. Green ∗, cell-dense area. Immunostaining for CTIP2 (COUP-TF-Interacting-Protein 2, green). Scale bar: 150 μm. G-H, SC102A at week 5, immunostained in green: G, PU.1 (hematopoietic transcription factor PU.1). H, RUNX1 (runt-related transcription factor 1). White arrow, overlap. Scale bar: 20 μm.

To identify whether 3D-retinal organoids contain microglia-like cells, we applied an established unguided retinal organoid differentiation protocol (Zhong et al., 2014) to two hiPSC lines of different origins (Figures 1A, S1A and S1B). One hiPSC line was derived from a 60^+^-old skin fibroblast donor (SC102A) and the other from fetal umbilical cord blood cells (CR05, Table S1). Both hiPSC lines behaved similarly and formed typical optic cup structures within four weeks in 2.5D culture. They developed further into anatomically comparable 3D-retinal cups (Figure S1C) expressing cell type-specific markers for photoreceptor-, bipolar-, amacrine-, ganglion, and Müller glial cells by week 18 (Figure S1D) (Hoshino et al., 2017; Luo et al., 2019; Zhang et al., 2019). We used IBA1 as a marker for microglia and confirmed the antibody functionality in human temporal lobe brain tissue, where IBA1 labeled parenchymal microglia and pvMΦ (Figure S2). When we immunostained our 3D-retinal organoids, we commonly observed no cell-defined IBA1 staining (Figure 1B). Occasionally, we found a few IBA1^+^-cells close to the retinal cup (Figure S3A), but the cells were not numerous or as deeply integrated as described for the human embryo retina at similar age (Diaz-Araya et al., 1995).

Based on this rare microglia presence, we hypothesized that IBA1^+^-cells might be enriched in a compartment other than the retinal cup. Thus, we revisited the 2.5D culture before dissection of optic cups at week 4 (Figure 1A). Between weeks 3 and 4, we found clusters of IBA1^+^-cells (Figure 1C), which started to spread within the culture by week 4 and occupy distinct compartments by week 7 (Figure 1D). These compartments were commonly less nuclei-dense (Figure 1E). To investigate whether these compartments contained cortical cell types, we stained the 2.5D culture with CTIP2/BCL11b (BAF chromatin remodeling complex subunit), a marker expressed in the neocortex from early embryonic stages (Qian et al., 2016). Remarkably, the majority of IBA1^+^-cells were distinct from the CTIP2^+^-region (Figure 1F), and if they were present, they mostly localized to the surface of these structures.

To confirm that IBA1^+^-cells were microglia-like, we immunostained the 2.5D cultures between week 4 and 5 for the hematopoietic lineage-specific markers RUNX1 (Ginhoux et al., 2010), PU.1 (Kierdorf et al., 2013), and MYB (Schulz et al., 2012). As expected, all IBA1^+^-cells were positive for RUNX1 and PU.1 and negative for MYB (Figures 1G, 1H, S3C, and S3D). The IBA1^+^-cells also expressed the mononucleate hematopoietic cell marker CD45 (Monier et al., 2007) (Figure S3E), the fractalkine receptor CX3CR1 (Hulshof et al., 2003) (Figure S3F), the purinergic receptor P2Y12 (Mildner et al., 2017) (Figure S3G) and did not express the monocytic marker CD14 (Geissmann et al., 2003) (Figure S3H). Importantly, all markers were cross-validated for their specificity in human brain tissue (Figure S2). The IBA1^+^-cells were morphologically branched and frequently presented phagocytic cups (Figure S3B). 47.9% +/− 5.7% of the IBA1^+^-cells co-expressed KI-67 indicating that they are in a proliferative state (Gerdes et al., 1984) (Figure S3I). The cells also expressed the mitotic marker phosphorylated histone H3 (PHH3) (Hirata et al., 2004) (Figure S3J). This characterization suggests that IBA1^+^-cells represent microglia-like cells that emerge within the unguided protocol in 2.5D culture by week four and, notably, do not extensively populate retinal cups or cerebral compartments.

### IBA1^+^-cells enrich in cystic compartments of 3D-aggregates

**Figure 2:**
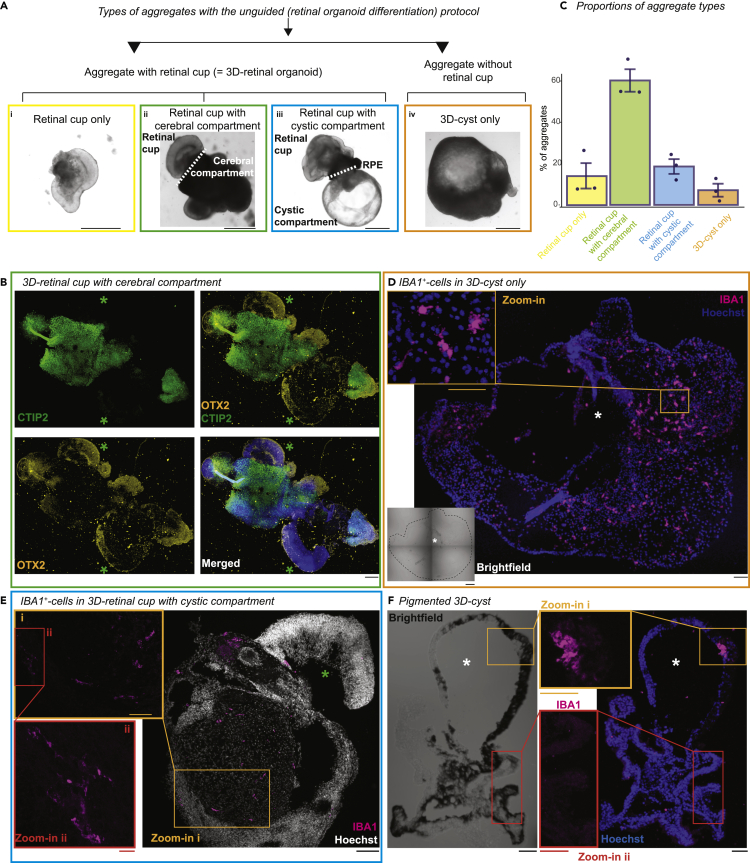
IBA1^+^-cells occupy 3D-cystic compartments (A) Representative bright field images of typical aggregates generated within unguided (retinal organoid differentiation) protocol at week 5 for SC102A. RPE: retinal pigment epithelium. Scale bar: 1000 μm. (B) Immunostaining of cryostat section of 3D-retinal cup with cerebral compartment (week 8-9) from SC102A stained for OTX2 (orthodenticle homeobox 2, orange) and CTIP2 (COUP-TF-Interacting-Protein 2, green) and nuclei stained with Hoechst (blue). Green ∗, retinal cup. Scale bar: 150 μm. (C) Mean percentage of aggregate type proportions with SEM. Each dot represents one differentiation. (D–F) Immunostaining of cryostat sections for IBA1 (ionized calcium-binding adapter molecule 1, magenta), nuclei stained with Hoechst (blue, except E in white). D, 3D-cyst from SC102A (week 8-9) with brightfield image. Dashed-line, cyst surrounding. White ∗, cystic lumen. Scale bar: 100 μm. Zoom-in to IBA1^+^-cells. Scale bar: 50 μm. (E) 3D-retinal organoid with cystic compartment for SC102A (9–10 weeks). Green ∗, retinal cup. Scale bar: 100 μm. Zoom-in: Scale bar: 50 μm (i), 10 μm (ii). (F) Pigmented 3D-cyst (week 8-9) from SC102A with bright field image. White ∗, cystic lumen. Scale bar: 100 μm, zoom-in: 50 μm.

The presence of IBA1^+^-cells in less nuclear-dense structures in 2.5D culture inspired us to revisit our 3D culture. We found that the unguided protocol results in two groups of aggregates: either with or without retinal cups (Figure 2A). The aggregates with retinal cups, summarized as 3D-retinal organoids, can represent either a retinal cup only (Figure 2A, i), a retinal cup with a cerebral compartment (Figure 2A, ii) that can be characterized with OTX2 (Orthodenticle Homeobox 2) and CTIP2 (Figure 2B), respectively (Hoshino et al., 2017; Qian et al., 2016), or a retinal cup with cystic compartment (Figure 2A, iii). We named an aggregate without a retinal cup a 3D-cyst (Figure 2A, iv). These cysts were semitransparent, contained various-sized lumens, and occasionally developed pigmentation or a cuboidal-shaped epithelial surface. We found approximately 10% of the aggregates to be non-retinal (Figure 2C), which is in line with previously reported studies (Cowan et al., 2020; Zhong et al., 2014). IBA1^+^-cells enriched and distributed in the 3D-cysts (Figure 2D) and the cystic compartment of retinal organoids (Figure 2E). Cystic compartments that were pigmented tended to lack IBA1^+^-cells (Figure 2F). This suggests that IBA1^+^-cells occurring with the unguided protocol preferentially occupy non-pigmented cystic compartments.

### Low-dosed BMP4 application enhances 3D-cysts and IBA1^+^-cells

**Figure 3:**
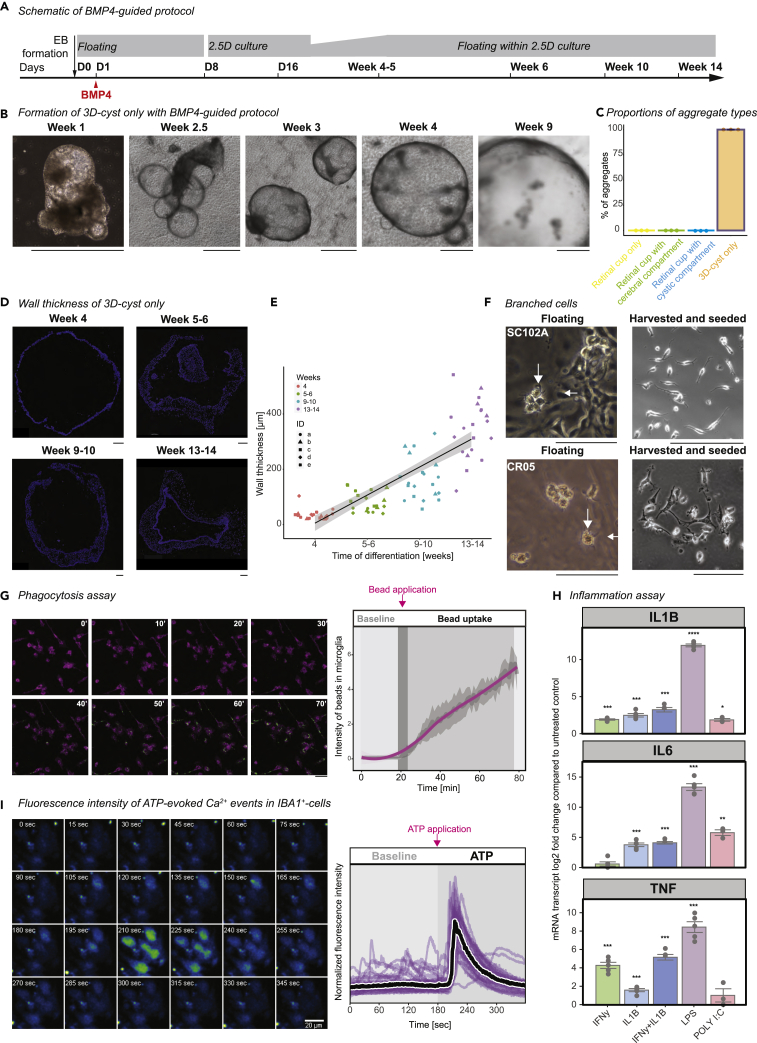
BMP4 induces 3D-cyst development (A) Schematic of guided differentiation protocol with a single BMP4 (bone morphogenetic protein 4) application on Day (D) 1 after induced differentiation. EB, embryoid bodies. (B) Brightfield images of developing 3D-cysts generated with BMP4-guided differentiation for SC102A. Scale bar: 1000 μm. (C) Mean percentage of aggregate type proportions generated using BMP4-guided protocol with SE. Each dot represents one differentiation. (D) Cryostat sections of 3D-cysts generated with BMP4-guided protocol at four different time points counter-stained with nuclei-dye Hoechst (blue). Scale bar: 100 μm. (E) Scatterplot of wall thickness. Each symbol (a-e) represents a 3D-cyst with four data points for the measured wall thickness on opposing sides. Five cysts per time point with trend curve and 95% confidence interval. (F) Brightfield images of branched cells in the supernatant for SC102A (top) and CR05 (bottom). Left, focus on floating cells in the original plate (week 6-7). Arrow, branching. Right, harvested supernatant and seeded on a new plate (week 5-6). Scale bar: 100 μm. (G) Phagocytosis assay. Left: Consecutive snapshots of live imaged tomato-lectin-labeled microglia-like cells (magenta) obtained from the supernatant from BMP4-guided protocol (SC102A) and their uptake of fluorescent beads (green) at week 6-7. Scale bar: 100 μm. Right: Mean intensity increase of beads within IBA1^+^-cells and 95% confidence interval band during 80 min of recording of three independent differentiations. Dark gray bar: bead application after 20 min of baseline recording. (H) Real-time quantitative PCR (RT-qPCR) for interleukin 1β (IL1β, top), interleukin 6 (IL6, middle), and tumor necrosis factor (TNF, bottom). HiPSC-derived microglia from BMP4-guided protocol were treated with either recombinant interferon γ (IFNγ), IL1β, or both, bacterial lipopolysaccharide (LPS), or polyinosinic:polycytidylic acid (poly I:C) at week 6-7. Bar chart: mRNA transcript log-2-fold changes compared to untreated control cells with SEM. Each dot represents an independent differentiation. One sample *t*-test. ∗p < 0.05, ∗∗p < 0.01, ∗∗p < 0.001 and ∗∗∗∗p < 0.0001. (I) ATP-evoked Ca^2+^ transients in IBA1^+^-cells derived from BMP4-guided protocol for SC102A at week 6-7. Left: Consecutive snapshots of live imaged cells exposed to Ca^2+^-sensitive fluorescent dye Fluo-4. Scale bar: 20μm. Right: Ca^2+^-dependent fluorescence intensity normalized to the mean intensity of the cells throughout 360 s of recording. After 180 s of baseline measurement (light gray area), ATP (1mM final concentration) was applied, and recording was continued up to 360 s (dark gray area). Each curve shows the Ca^2+^-events of an individual cell. Black line: Median of 32 cells from three independent differentiations.

Recent hiPSC-derived microglia-like protocols have reported that bone morphogenetic protein 4 (BMP4) promotes microglia generation *in vitro* (Abud et al., 2017; Douvaras et al., 2017; Guttikonda et al., 2021; Haenseler et al., 2017; Pandya et al., 2017; Takata et al., 2017) with some studies mentioning the development of cystic structures (Haenseler et al., 2017; Muffat et al., 2016; Vaughan-Jackson et al., 2021). Because we sought insight into the tissue identity of the IBA1^+^-cell enriched cystic compartments and we were confronted with the heterogeneity of our unguided protocol culture, we decided to enrich for the cystic compartments with a low-dosed BMP4 application one day after EB formation to the otherwise unchanged protocol (Figure 3A). After BMP4 exposure, EBs formed an irregular shape and developed non-pigmented 3D-cysts that started to float by week 3 (Figures 3B, S4A). These 3D-cysts were the only aggregates formed by the BMP4-guided protocol in both hiPSC lines (Figure 3C). They neither expressed recoverin nor BRN3 for labeling photoreceptors and retinal ganglion cells, respectively (Figures S4B and S4C), nor the neuronal marker beta-III-tubulin (Figure S4D). OTX2 was expressed by week nine but did not show an overlap with recoverin, both labeling photoreceptors (Figure S4B). This supports the previous observation with BMP4 to induce mesoderm (Faial et al., 2015; Zhang et al., 2008). The lack of neuroectoderm is already prevalent at Day 12, when the formation of neuronal filaments is absent upon BMP4 application (Figures S4E and S4F). These 3D-cysts gradually grew in diameter and expanded their inner wall thickness (Figure 3D and 3E).

From week 4 onward, small branched cells started to float in the supernatant (Figure 3F), which have previously been described as microglia-like cells (Haenseler et al., 2017). To verify this cell identity, we either collected and seeded these cells or directly labeled them on 2.5D culture plates (Figure S5). In both cases, the IBA1^+^-cells expressed RUNX1, PU.1, CD45, and P2Y12 but not CD14 (Figures S5A–S5H). Moreover, we confirmed their mRNA expression for PU.1, IBA1, P2Y12, and CX3CR1 with real-time qPCR (RT-qPCR, Figure S5I), suggesting that they are microglia-like cells similar to the microglia obtained with the unguided protocol (Figures 1G, 1H, and S3C–S3H).

To further validate a microglia-like activity, we first measured the capability of IBA1^+^-cells to phagocytose. We immunostained the IBA1^+^-cells with tomato-lectin and live imaged the uptake of pH-sensitive fluorescent beads. The beads accumulated overtime within the IBA1^+^-cells indicating phagocytic active cells (Figures 3G and S6A). Next, we investigated whether the IBA1^+^-cells trigger an upregulation of inflammatory signature genes IL6, TNF, and IL1β (Smith et al., 2012) upon stimulation with either interferon γ (IFNγ), interleukin 1β (IL1β) or both bacterial lipopolysaccharide (LPS) or poly I:C. For all stimulations, we confirmed the upregulation of these inflammatory genes in IBA1^+^-cells (Figure 3H). Finally, to test whether hiPSC-derived IBA1^+^-cells display Ca^2+^-events upon extracellular ATP administration (Palomba et al., 2021), we labeled IBA1^+^-cells with the Ca^2+^-sensitive fluorescent dye Fluo-4 and imaged the fluorescent intensity after ATP administration. IBA1^+^-cells displayed rapid and synchronized accumulation of Ca^2+^-events (Figure 3I), which was not observed with medium-treated IBA1^+^-cells (Figures S6B and S6C).

### IBA1^+^-cells populate but do not originate in 3D-cysts in BMP4-guided protocol

**Figure 4:**
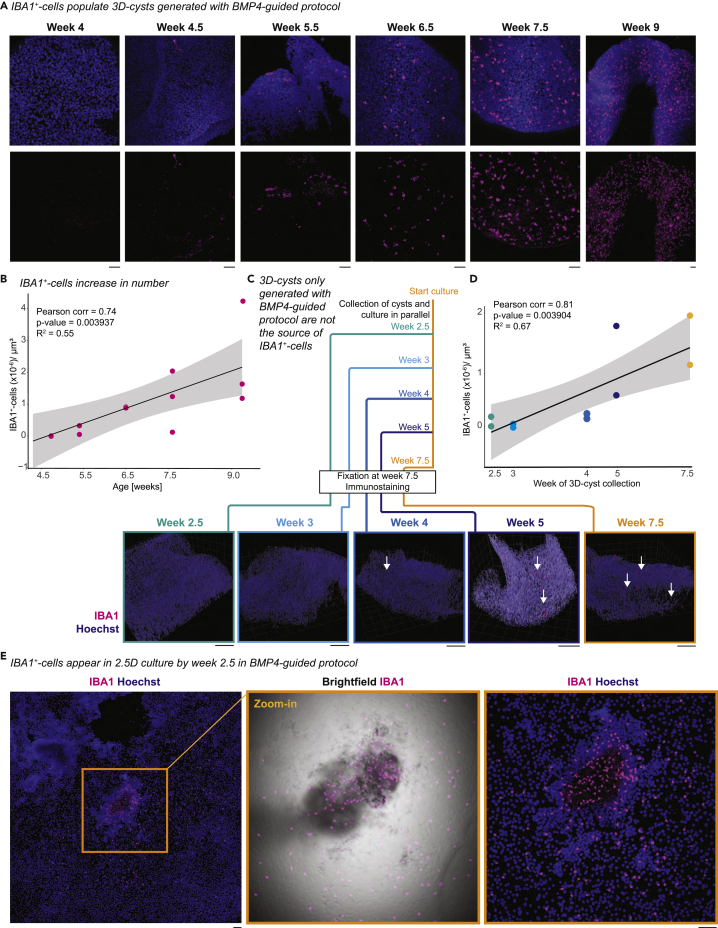
IBA1^+^- populate but do not originate in 3D-cysts in BMP4-guided culture Immunostaining for IBA1 (ionized calcium-binding adapter molecule 1, magenta) and Hoechst (blue) in SC102A for BMP4-guided protocol. (A) Timeline of the presence of IBA1^+^-cells in 3D-cysts from week 2.5 to week 9. Scale bar: 50 μm. (B) Scatterplot of IBA1^+^-cells occupying 3D-cyst with trend curve and 95% confidence interval. Pearson correlation showing a significant correlation between age of differentiation and number of IBA1^+^-cells occupying the cyst (Pearson’s correlation = 0.738518 and p value = 0.003937, R^2^ = 0.5454089). (C) 3D-cysts collected and separated from the 2.5D culture at week 2.5, 3, 4, five, and cultured until week 7.5, immunostained (wholemount) together with 3D-cysts which were kept in the original 2.5D culture until week 7.5 (orange frame). Representative images of 3D volume rendering to visualize the 3D-cyst with IBA1^+^-cells. White arrow, IBA1^+^-cells. Scale bar: 100 μm. (D) Scatterplot of IBA1^+^-cells occupying isolated 3D-cyst with trend curve and 95% confidence interval. Pearson correlation showing a significant correlation between age when 3D cysts were isolated and IBA1^+^-cells density (R = 0.81707 and p value = 0.0039, R^2^ = 0.6676). (E) First occurrence of IBA1^+^-cells in 2.5D culture. Orange frame, zoom-in with brightfield image. Scale bar: 100 μm.

To identify whether IBA1^+^-cells, similarly to the unguided protocol, occupy 3D-cysts in BMP4-guided protocol, we collected 3D-cysts at several time points after differentiation and performed wholemount immunostaining. Starting at week 5.5, IBA1^+^-cells populated the 3D-cysts and increased in number over time (Figures 4A and 4B). To identify whether 3D-cysts might be the source of IBA1^+^-cells, we collected 3D-cysts from a 2.5D culture plate at week 2.5, 3, 4, and five and cultured these separately, in parallel to the left-over 3D-cysts. These 3D-cyst cultures were developed until week 7.5 and then immunostained for IBA1 (Figure 4C). Unexpectedly, the 3D-cysts isolated at week 2.5–four contained only a few IBA1^+^-cells (Figure 4D). In contrast, 3D-cysts isolated at week five had a similarly high number of IBA1^+^-cells to the 3D-cysts cultured on the original plate excluding these structures as the source of IBA1^+^-cells. Indeed, we found IBA1^+^-cells already on the culture plate between week two and 3 (Figure 4E) indicating that they are not derived from the 3D-cysts.

### IBA1^+^-cells associate with the mesenchymal/vimentin^+^-region

**Figure 5:**
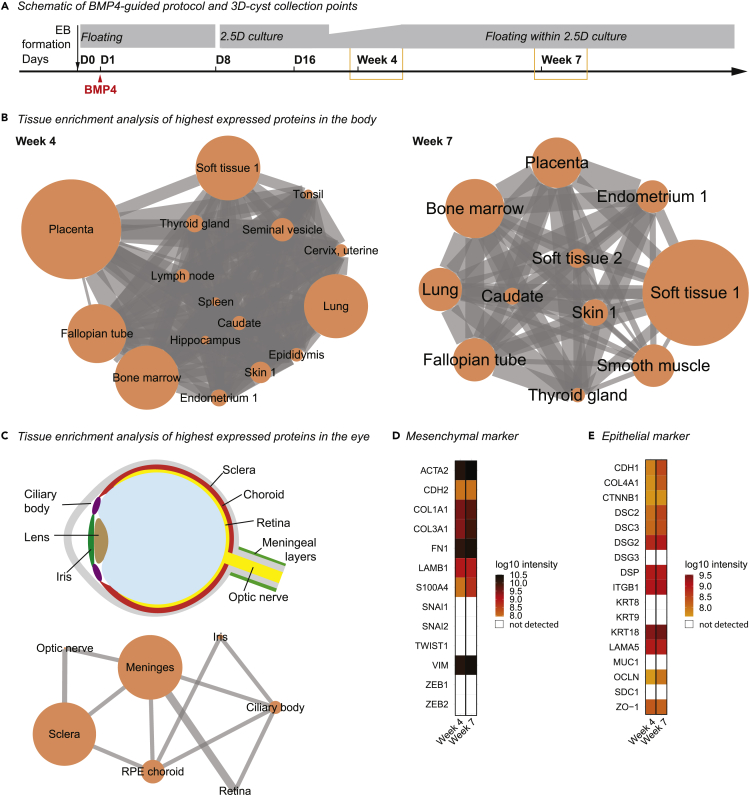
Tissue-specific protein enrichment analysis of 3D-cysts indicates a mesenchymal-like compartment (A) Schematic of experimental design. For mass spectrometry, ten 3D-cysts generated with BMP4-guided protocol were collected at week 4 and 7. (B) Tissue enrichment analysis shown as a network. Nodes, tissue. Edges, connecting the tissues that share highly expressed proteins in the 98th percentile. The node size reflects the enrichment p value and the thickness of the edge reflects the size of the shared proteins (see also: Table S4). (C) Top, eye schematic. Bottom, tissue enrichment analysis of the week seven dataset. RPE, retinal pigment epithelium (see also: Table S5). (D and E) Heatmap of protein expression level in log10 (intensity) for week 4 (W4) and week 7 (W7) cystic compartments for D, mesenchymal marker (see also: Table S6); E, epithelial marker (see also: Table S6).

To obtain insights into the cyst composition, we performed mass spectrometry of 3D-cysts from the BMP4-guided protocol with either sparse or high IBA1^+^-cell population at week 4 and 7, respectively (Figure 5A). We obtained the peptide sequence data (Table S2) and compared the highly expressed proteins with 44 tissues from the human proteome atlas that have previously been characterized (Uhlén et al., 2015). At week 4, highly abundant proteins suggest that cells in the 3D-cysts have various fate potentials (Figure 5B, Tables S3 and S4). Interestingly, at week 7, the protein composition was specific to tissues of partial or full mesodermal origin such as soft tissue, bone marrow, and smooth muscles, which is consistent with BMP4 application and indicates a mesenchymal identity (Figure 5B, Tables S3 and S4). Similarly, when we performed tissue enrichment analysis of proteins related to human eye tissue (Dunn et al., 2019; Zhang et al., 2015, 2016a), we found a significant overlap of highly abundant proteins for meninges and sclera, both of which have mesenchymal origin. In contrast, proteins enriched in the ectodermal retina and optic nerve were underrepresented (Figure 5C and Table S5).

**Figure 6:**
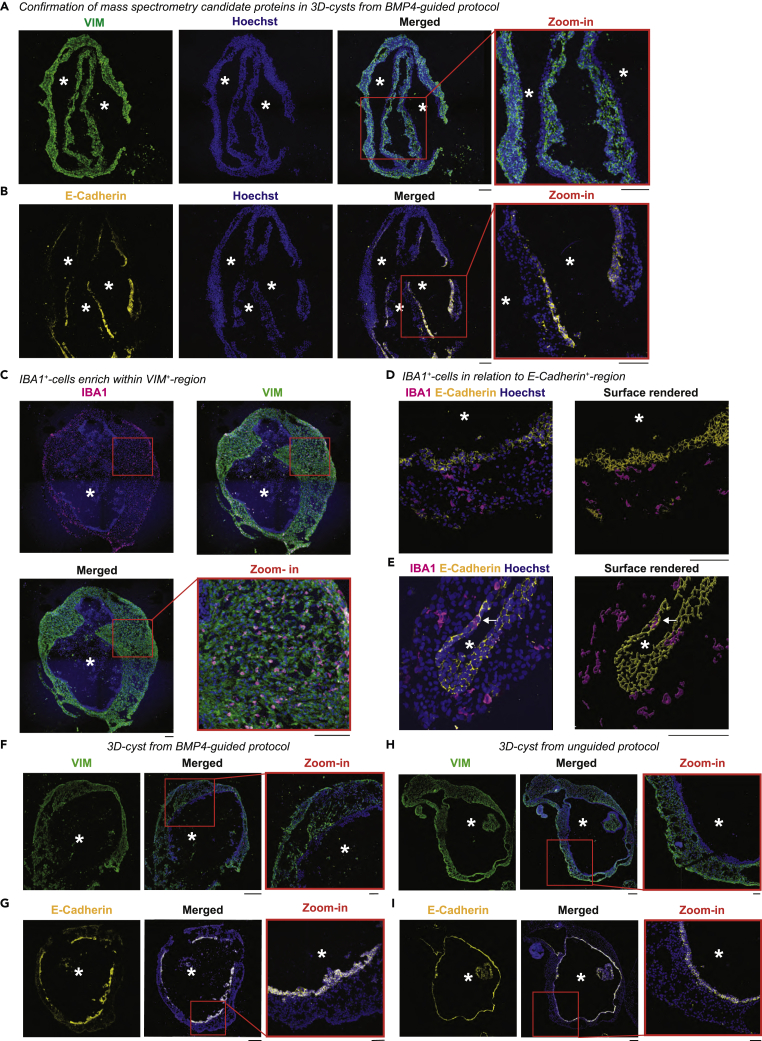
IBA1^+^-cells localize within VIM^+^-region in cystic compartment Immunostaining of cryostat sections of 3D-cysts from BMP4-guided (A–G) or unguided (H and I) protocols, counter-stained with the nuclei-dye Hoechst (blue). ∗, lumen. (A–E) Scale bar: 100 μm. (A and B) Sequential sections of SC102A cysts stained with VIM (vimentin, green, A) and E-Cadherin (yellow, B) at week 7 with zoom-in (orange frame). (C–E) Staining for IBA1 (ionized calcium-binding adapter molecule 1, magenta), VIM (vimentin, green, week 12, C), and E-Cadherin (yellow, week 10, with 3D-surface rendering for CR05 cysts D-E). White arrow, IBA1^+^-cells within the E-Cadherin layer. (F–I) Comparison of immunostaining of VIM (vimentin, green, F, H) and E-Cadherin (yellow, G, I) for SC102A at week eight for 3D-cysts from BMP4-guided (F-G) and unguided (H-I) protocol. Scale bar: 200 μm. Zoom in, Scale bar: 50 μm.

To validate whether the 3D-cysts are enriched for mesenchyme, we compared our mass spectrometry data with mesenchymal markers including transcription factors, cytoskeletal-, cell surface, and extracellular matrix proteins (Andrzejewska et al., 2019; Owusu-Akyaw et al., 2019; Scanlon et al., 2013). We found that vimentin (VIM), laminin β1 (LAMB1), and fibronectin (FN1) were enriched, with VIM among the most abundantly expressed mesenchymal proteins at week 7 (Figure 5D). Because one characteristic of mesenchyme is close interaction with the epithelium (MacCord, 2012), we also investigated the presence of epithelial proteins in our dataset and found several to be upregulated from week four–7 (Figure 5E). One of these epithelial markers is E-Cadherin (CDH1), which is commonly described together with the mesenchymal marker VIM in the epithelial-mesenchymal transition during development and cancer (Hay, 2005; Thiery et al., 2009; Yamashita et al., 2018). Therefore, we validated the expression of both markers in 3D-cysts derived with the BMP4-guided protocol. VIM expression was strong in the cell layers facing the surface of the 3D-cyst wall (Figure 6A). In contrast, E-Cadherin marked a defined layer next to the cystic lumen and complemented VIM expression (Figure 6B). When we investigated the location of IBA1^+^-cells, they mostly occupied the VIM^+^-region (Figure 6C) and stayed distinct from the E-Cadherin^+^-layer (Figure 6D). Only occasionally IBA1^+^-cells intermingled with the E-Cadherin^+^-cells (Figure 6E).

**Figure 7:**
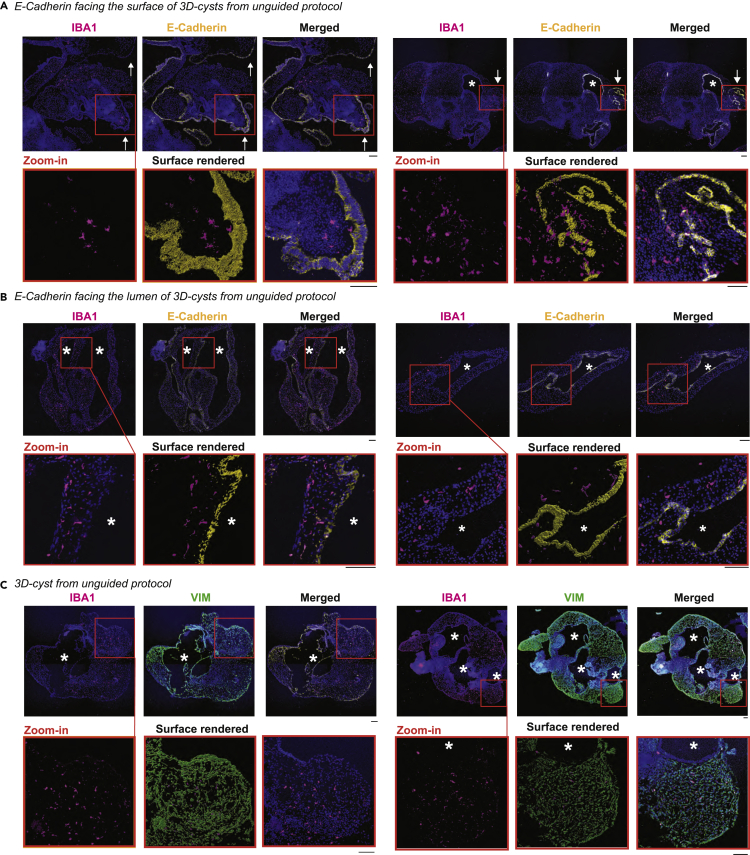
IBA1^+^-cells preferentially localize within VIM^+^-regions in 3D-cysts Immunostaining of cryostat sections of 3D-cysts from unguided protocol for IBA1 (ionized calcium-binding adapter molecule 1, magenta), E-Cadherin (yellow, A and B), VIM (vimentin, green, C), and the nuclei-dye Hoechst (blue) for SC102A at week 8. ∗, lumen within the cystic compartment. A, Arrow, E-Cadherin staining on 3D-cyst surface. Red frame, zoom-in with surface rendering in the middle. Scale bar: 100 μm.

To verify whether the IBA1^+^-cells prefer the mesenchymal-like VIM^+^-region also in 3D-cysts obtained from the unguided protocol (Figure 1A), we repeated the aforementioned staining. VIM labeled a similar defined region in the 3D-cysts with E-Cadherin expression localized in a defined layer around the cystic lumen (Figures 6F–6I). In addition, we found E-Cadherin^+^-expression at regions facing the surface (Figure 7A). IBA1^+^-cells rarely intermingled with the E-Cadherin^+^-layer facing the lumen or the surface of the 3D-cyst (Figures 7A and 7B) and mostly localized within the VIM^+^-region (Figure 7C) suggesting that IBA1^+^-cells prefer the mesenchymal region.

Mesenchymal stem cells can have immunomodulatory capabilities (Andrzejewska et al., 2019) and could sequester IBA1^+^-cells away from infiltrating into the retinal cups. To test this, we added 17-week-old retinal organoids without a cystic compartment (Figure 2A, i/ii) from the unguided protocol to the culture of BMP4-guided protocol, which contains both floating IBA1^+^-cells and 3D-cysts in the supernatant (Figure S7A). IBA1^+^-cells did not integrate into the retinal cup or the cerebral compartment (Figure S7B). In contrast, if we harvested IBA1^+^-cells from the BMP4-guided protocol and applied them to 3D-retinal organoids without a cystic compartment (Figure S7C), IBA1^+^-cells successfully integrated into both retinal cup and cerebral compartment (Figures S7D and S7E). These data show that when there is no cystic compartment, IBA1^+^-cells start to occupy the retinal cup. Otherwise, they are preferentially enriched in the mesenchymal, cystic region.

### IBA1^+^-cells adopt a BAM signature in the mesenchymal environment

**Figure 8:**
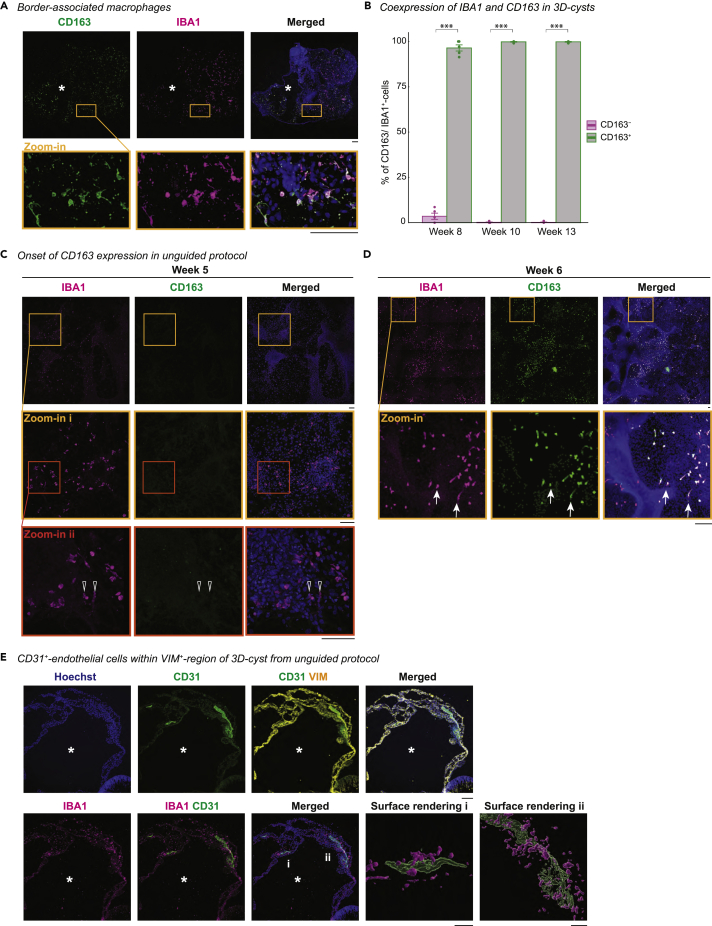
IBA1^+^-cells express CD163, a marker for border-associated macrophages Immunostaining of cryostat sections of SC102A 3D-cysts from unguided protocol for IBA1 (ionized calcium-binding adapter molecule 1, magenta) and the nuclei-dye Hoechst (blue). ∗, lumen within the cystic compartment. (A) Immunostaining of CD163 (cluster of differentiation 163/protein tyrosine phosphatase receptor type C, green) in SC102A at week 8 with zoom-in (Orange frame). Scale bar: 100 μm. (B) Bar chart of % of CD163^+^/IBA1^+^-cells and % of CD163^-^/IBA1^+^-cells per time point with SE. Each dot represents one section of an individual 3D-cyst. two-way ANOVA p value = < 2.2e-16 with selected post hoc test. ∗∗∗p < 0.001. (C and D) CD163 expression in 2.5D-culture for CR05 at week 5 (C) and week 6 (D). Open arrow, CD163^-^/IBA1^+^. Arrow, CD163^+^/IBA1^+^. Red frame, zoom in. Scale bar: 100 μm. (E) Immunostaining for CD31 (platelet and endothelial cell adhesion molecule 1, green) and VIM (vimentin, yellow) for SC102A at week 8. Scale bar: 100 μm. Zoom-in, 3D-surface rendering (i, ii). Scale bar: 20 μm.

Border-associated macrophages (BAMs) are non-parenchymal macrophages that reside either at perivascular structures, meninges, or choroid plexus, all of mesenchymal nature (Lun et al., 2015; MacCord, 2012; Pill et al., 2015; Wimmer et al., 2019). Transcriptional profiling of macrophage populations in embryonic mouse brain identified CD163 as a potential marker for BAMs (Utz et al., 2020), which also labels human perivascular macrophages (Fabriek et al., 2005), mononuclear phagocytes in the choroid plexus, and cells in the meningeal- and subpial granular layer (Rezaie and Male, 2003). Indeed, we found that 99% of IBA1^+^-cells co-expressed CD163 by week 10 in the 3D-cyst (Figures 8A and 8B). Interestingly, the onset of CD163 expression occurs in a defined window. At week 5, IBA1^+^-cells were still negative for CD163 in the 2.5D culture (Figure 8C). Within one week, IBA1^+^-cells co-expressed CD163, as they started to distribute within the 2.5D culture and occupy compartments that were sparse in nuclei (Figure 8D). To identify whether the 3D-cysts expressed a blood vessel endothelium, we stained for CD31. Only occasionally, we observed CD31 in the VIM^+^-region (Figure 8E), suggesting that IBA1^+^-cells occupy the 3D-cyst even without a blood vessel system. In contrast to mice that require the blood vessel system (Ginhoux et al., 2010), human microglia infiltrate the cortex from the ventricular lumen and the leptomeninges (Monier et al., 2007; Rezaie et al., 2005); thus, the strong preference of IBA1^+^-cells to the mesenchyme might explain the preferential location of IBA1^+^-cells to infiltrate the brain tissue.

## Discussion

In this study, we demonstrate that microglia-like cells emerge between week 3 to 4 in 2.5D culture (Figure 1C) in an unguided retinal organoid differentiation protocol (Zhong et al., 2014). At this time point, the 2.5D culture is highly heterogeneous and reflects an unperturbed self-organized environment. The properties of EBs in our unguided protocol allow the formation of retinal cell types derived from the neuroectodermal lineage (Figure S1D) and mesenchymal cells that are mainly derived from the mesoderm (Figures 6H, 7C). This environment and the time frame is similar to when IBA1^+^-cells have been reported to appear in human embryonic development (Bloom and Bartelmez, 1940; Kelemen and Jánossa, 1980; Monier et al., 2007; Rezaie et al., 2005). Our results also support a recent study that identified a cluster of microglial cells using RNA-sequencing of cerebral organoids with bilateral optic vesicles (Gabriel et al., 2021). However, in contrast to human embryonic retinal development (Hu et al., 2019), we rarely observed microglia-like cells in the hiPSC-derived retinal cups at 5 weeks or later (Figure S3A). Instead, IBA1^+^-cells strongly preferred the mesenchymal-like cystic over the neuronal compartment (Figures 1E and 1F, 2).

In humans, microglia first enter the embryonic cortical regions via the ventricular lumen, choroid plexus, and leptomeninges (Menassa and Gomez-Nicola, 2018; Monier et al., 2007; Rezaie and Male, 2003)—all tissues originating from the mesenchyme (Catala, 2019; Lopes, 2009; O’Rahilly and Müller, 1986). As mesenchymal structures develop *in vivo* around the neuronal retina with the choroid close to the photoreceptors and meninges wrapping the optic nerve (Forrester et al., 2010; Sturrock, 1987), the preferential location of our IBA1^+^-cells in the mesenchymal region potentially recapitulates how microglia enter the retina. The CD163 expression of IBA1^+^-cells further supports a perivascular-associated role. Initially, parenchymal microglia and border-associated macrophages are derived *in vivo* from the same primitive macrophage precursor (Goldmann et al., 2016) and then adapt their transcriptional landscape to their local environment at early developmental stages (Gosselin et al., 2017; Masuda et al., 2019; Utz et al., 2020). CD163 is one example of a human border-associated- (perivascular, leptomeningeal, choroid plexus) macrophage marker (Fabriek et al., 2005; Rezaie and Male, 2003), and its expression is upregulated during mouse embryonic development (Utz et al., 2020). Indeed, our IBA1^+^-cells in the 2.5D culture arose as CD163 negative and expressed CD163 after one week (Figures 8C and 8D).

### Comparison of microglia occurrence between Shiraki et al. 2022 and our study

A recent study by Shiraki et al. (2022) describes that PAX6-positive microglia evolved in hiPSC-derived ocular organoids (Shiraki et al., 2022). The authors took advantage of their recently developed ‘SEAM’ (self-formed ectodermal autonomous multizone) protocol, which provides the potential to differentiate hiPSCs into anlages of different ocular lineages such as neuroectoderm (zone 1), neural crest (zone 2), ocular-surface ectoderm (zone 3), or non-ocular surface ectoderm (zone 4) (Hayashi et al., 2016, 2017). Their recent paper found that microglia-like cells occur between zone 2–3 as early as day 10 after the hiPSC differentiation (Shiraki et al., 2022). To further validate the putative microglia-like cells, they performed immunostainings for TMEM119, CX3CR1, and CD11b. Moreover, they performed RT-qPCR analysis from SEAM for selected microglia gene candidates and single cell-RNA sequencing of CD11b^+^/CD45^low^-cells at week 4.

In our study, we have differentiated retinal organoids with a modified protocol by Zhong et al. (2014) and used embryoid bodies seeded on day 8 on Matrigel (Zhong et al., 2014). After week 4, optic cups are cut out and then cultured in suspension to allow the maturation of 3D-retinal organoids or alternative aggregates as we have described in this manuscript (Figure 2A). In contrast to Shiraki et al. (2022), we identified microglia-like cells with IBA1. IBA1 is a well-established marker commonly used for hiPSC-derived microglia-like cells (Abud et al., 2017; Douvaras et al., 2017; Haenseler et al., 2017; McQuade et al., 2018; Muffat et al., 2016; Pandya et al., 2017) and has not been used for immunostaining by Shiraki et al. (2022). Furthermore, IBA1 has been shown to be highly specific in human embryonic tissue at gestation week 4.5 (Monier et al., 2007). We found the earliest expression of IBA1^+^-cells between week three to four in our 2.5D culture (Figure 1C), which is in line with observations from Ormel et al. (2018), who describes the first IBA1^+^-cells at day 24 (Ormel et al., 2018).

We validated that our IBA1^+^-cells express microglia-markers such as RUNX1, PU.1, CD45, CX3CR1, and P2Y12 (Figures 1G and 1H, 3C, 3E–3G, and S5) and the mRNA of PU.1, IBA1, CX3CR1, and P2Y12 (Figure 5I). Furthermore, our IBA1^+^-cells demonstrated the expected phagocytic capability (Figures 3G, S6A)—an inflammatory signature upon stimulation (Figure 3H)—and calcium signaling response upon extracellular ATP exposure (Figure 3I) confirming their microglia-like identity. When we compared the images of the TMEM119-positive microglia-like population to our IBA1^+^-cells, we were surprised about the size of more than 200 μm and their highly branched morphology at week 4. Both parameters are rather unusual: First, in the adult human brain tissue, the average size of TMEM119^+^/IBA1^+^-immunostained microglia is around 50 μm (Figure S8A). Second, in human embryonic brain tissue, the average size of microglia stained with CD68, IBA1, or CD45 is around 20 μm (Monier et al., 2007; Rezaie and Male, 2003). Similarly, our hiPSCs-derived microglia cell diameter ranges from 20 to 100 μm (Figures S3, S5A–S5H), which is in line with previous studies (Abud et al., 2017; Douvaras et al., 2017; Haenseler et al., 2017; McQuade et al., 2018; Muffat et al., 2016; Ormel et al., 2018; Pandya et al., 2017; Takata et al., 2017). Finally, we and others identified microglia to typically reflect a more amoeboid or bipolar-shaped morphology during human embryonic development (Cunningham et al., 2013; Diaz-Araya et al., 1995; Monier et al., 2007; Rezaie et al., 2005). We are not aware of another study that shows a similar highly branched microglia-like cell network starting from week 2 as described by Shiraki et al. (2022). Even in adulthood, individual microglia rarely overlap with their processes (Figure S2).

Therefore, we decided to stain our 2.5D culture for TMEM119 at week four to compare the expression pattern with Shiraki et al. (2022). TMEM119 accumulated similarly around the retinal cup and the cells showed a radial distribution (Figure S8B). The approximate size of TMEM119^+^-cells is around 200 μm as described in Shiraki et al. and rather be atypical for microglia. Moreover, we found that cell type-specific TMEM119 staining also occurred at the 3D-cyst (Figure S8C). Because we have shown that IBA1^+^-cells specifically enrich at the 3D-cyst, we stained for IBA1. Surprisingly, IBA1^+^-cells neither overlap with TMEM119 at week 4.5 (Figure S8D) nor at week 8.5 (Figure S8E). Instead, TMEM119 and IBA1 labeled distinct cells, which were located next to each other. We validated two TMEM119 antibodies (Abcam, ab185333, polyclonal, binding at the C-terminus, and Novus Biologicals, NBP2-30551, polyclonal) with the Abcam antibody overlapping the antibody peptide sequence from the Sigma HPA051870 polyclonal antibody used by Shiraki et al. (2022). For both antibodies, we observed the same picture.

TMEM119 has been described as a selective marker for both human and mouse parenchymal microglia/IBA1^+^-cells (Bennett et al., 2016). However, this assumption has been recently challenged: For example, studies have shown that TMEM119 is neither exclusive to microglia nor does it label all microglia (Satoh et al., 2016; Vankriekelsvenne et al., 2022).

In addition, the specificity of TMEM119 to microglia-precursors during development is unclear. TMEM119 (type IA single-pass transmembrane protein) is also known as osteoblast induction factor Obif, which has a reported role in osteoblast differentiation and bone development (Jiang et al., 2017; Kanamoto et al., 2009; Mizuhashi et al., 2012; Tanaka et al., 2014). Thus, the observed TMEM119 staining might not necessarily reflect microglia. The additional represented markers CD11b (ITGAM) and CX3CR1 are commonly used for labeling microglia, but both have been also associated with regulation of bone homeostasis and osteoblasts (Hoshino et al., 2013; Koizumi et al., 2009; Park-Min et al., 2013), which could potentially explain their TMEM119 co-expression.

The performed RT-qPCR for other microglia markers by Shiraki et al. (2022) shows a high SD in the first 2–3 weeks and an overall low expression value suggesting low abundance. Only by week 4, they start to see a stronger signal, which is also the time point where they enrich for CD11b^+^/CD45^+^-cells for single-cell RNA sequencing. The switch to enrich for CD11b^+^/CD45^+^ and not TMEM119^+^-cells at week 4 will likely explain their success to find microglia-like transcriptional signatures. Week 4 is also the time point when we reliably observed IBA1^+^-cells (Figures 1D) and Ormel et al. (2018) showed that their microglia express CD11b and CD45.

Overall, we are convinced that the TMEM119 staining in Shiraki et al. (2022) does not represent microglia-like cells based on the discrepancy outlined before about the microglia phenotypes, our independently performed TMEM119 staining, and a potential alternative explanation of the role of TMEM119 during development, which might have been overshadowed by the preferential association of TMEM119 being a microglia-selective marker. Future studies will have to investigate whether TMEM119 reflects a human developmental signature gene for early microglia precursor cells.

In summary, our study confirms that microglia-like cells occur alongside human iPSC-derived retinal organoids during unguided differentiation and preferentially occupy the mesenchymal region. These findings will allow future analysis of microglial migration in complex tissue environments and facilitate identification of mechanistic cues that attract microglia in complex tissue structures.

### Limitations of the study

It remains unclear why IBA1^+^-cells do not further infiltrate the neuronal compartment especially when 3D-aggregates contain both retinal cups and cystic compartments (Figure 2A/iii, 2E). Even if we transferred 3D-retinal organoids to 2.5D cultures of BMP4-guided protocol, IBA1^+^-cells favor the cystic over the neuronal compartment (Figure S7). It is possible that the cystic compartment releases guidance signals that attract IBA1^+^-cells. These cues are likely to be similar to those that recruit macrophages to the epithelial-mesenchymal transition sides in glioma (Song et al., 2017). There is limited knowledge about developmental guidance cues that attract microglia to the neuronal compartment and more specifically to the retina. Further investigation will be required to answer these questions.

Furthermore, we employed two hiPSC lines from different origins and both resulted in a similar phenotype. We cannot exclude that the qualitative outcome could be different for other hiPSC lines because of properties related to genetic origin, epigenetic landscape, or transcriptional state at the time of the differentiation (Kilpinen et al., 2017; Ortmann and Vallier, 2017). Such factors might prevent the generation of cystic compartments and therefore the appearance of microglia-like cells.

## STAR★Methods

### Key resources table

**Table:** 

REAGENT or RESOURCE	SOURCE	IDENTIFIER
**Antibodies**		

Mouse monoclonal anti-Beta-III-tubulin (clone SDL.3D10), 1:100	Sigma-Aldrich	Cat#T8660-100UL; RRID: AB_477590
Goat polyclonal anti-BRN3 (clone C-13), 1:100	Santa Cruz Biotechnology	Cat#sc-6026; RRID: AB_673441
Goat polyclonal anti-CD14, 1:100	LifeSpan Biosciences	Cat#LS-B3012; RRID: AB_1965166
Rabbit polyclonal anti-CD31, 1:50	Abcam	Cat#ab28364; RRID: AB_726362
Rabbit monoclonal anti-CD45 (clone D9M8I), 1:200	Cell Signaling Technology	Cat#13917P; RRID: AB_2750898
Mouse monoclonal anti-CD163 (clone EDHu-1), 1:100	Bio-Rad AbD Serotec GmbH	Cat#MCA1853; RRID: AB_2074540
Goat polyclonal anti-ChAT, 1:400	EMD Millipore	Cat#AB144P; RRID: AB_2079751
Mouse monoclonal anti-CRALBP (clone B2), 1:200	Abcam	Cat#ab15051; RRID: AB_2269474
Mouse monoclonal anti-CtBP2 (clone 16), 1:200	BD Biosciences	Cat#612044; RRID: AB_399431
Rat monoclonal anti-CTIP2 (clone 25B_6_), 1:100	Abcam	Cat#ab18465; RRID: AB_2064130
Mouse monoclonal anti-CX3CR1 (clone K0124 × 10^1^), 1:50	BioLegend	Cat#B355702; RRID: AB_2561726
Mouse monoclonal anti-E-Cadherin (Clone 36/E-Cadherin), 1:100	BD Biosciences	Cat#610182; RRID: AB_397581
Rabbit polyclonal anti-IBA1, 1:750	GeneTex	Cat#GTX100042; RRID: AB_1240434
Goat polyclonal anti-IBA1, 1:250	Abcam	Cat#ab5076; RRID: AB_2224402
Mouse monoclonal anti-KI67 (Clone B56), 1:100	BD Biosciences	Cat#550609; RRID: AB_393778
Mouse polyclonal anti-MYB, 1:100	Acris	Cat#AP31223PU-N; RRID: AB_10976997
Goat polyclonal anti-OTX2, 1:150	R&D Sysytems	Cat#AF1979; RRID: AB_2157172
Rabbit polyclonal anti-P2Y12, 1:100	Sigma-Aldrich	Cat#HPA014518; RRID: AB_2669027
Mouse monoclonal anti-PAX6 (Clone PAX6/496), 1:400	Acris Antibodies/EuBIO Koeck	Cat#AM50305PU-N; RRID: AB_2895216
Rabbit polyclonal anti-PHOSPHO HISTONEH3 (PHH3), 1:300	Merck Millipore	Cat#06–570; RRID: AB_310177
Rabbit polyclonal anti-PU.1, 1:500	Cell Signaling Technology	Cat#2266S; RRID: AB_10692379
Rabbit polyclonal anti-Recoverin, 1:400	EMD Millipore	Cat#AB5585; RRID: AB_2253622
Mouse monoclonal anti-RUNX.1 (Clone 1C5B16), 1:50	BioLegend	Cat#659302; RRID: AB_2563194
Rabbit polyclonal anti-TMEM119, 1:100	Abcam	Cat#ab185333; RRID: AB_2687894
Rabbit polyclonal anti-TMEM119, 1:100	Novus Biologicals	Cat#NBP2-30551; RRID: AB_2910564
Mouse anti-VIMENTIN, 1:100	Santa Cruz Biotechnology	Cat#sc-6260; RRID: AB_628437

**Chemicals, peptides, and recombinant proteins**		

Blebbistatin	Sigma	Cat#B0560-5MG
N2 supplement	Gibco	Cat#17502-48
B27 without vitamin A	Thermo Fisher Scientific	Cat#121587-10
Retinoic acid	Sigma-Aldrich	Cat#R2625
Human BMP4	Peprotech	Cat#120-05
Human IFN-γ	Sigma-Aldrich	Cat#SRP3058-100UG
Human IL1-β	Thermo Scientific	Cat#RIL1BI
Lipopolysaccharid (LPS)	Sigma-Aldrich	Cat#L5886-10MG
Poly (I:C)	Tocris Bioscience	Cat#4287

**Critical commercial assays**		

innuPREP RNA Mini Kit 2.0	Analytik-Jena	Cat#845-KS-2040050
LunaScript RT Super-Mix Kit	New England Biolabs	Cat#E3010L
Luna Universal qPCR Master Mix	New England BioLabs	Cat#M3003L
in-Stage Tips kit	Preomics	P.O.00001

**Experimental models: Cell lines**		

Human: iPSC cell line SC 102A-1	BioCat	hPSCreg.eu: SBLi006-A
Human: iPSC cell line NCRM-5 (CR0000005)	RUCDR Infinite Biologics	hPSCreg.eu: CRMi001-A

**Oligonucleotides**		

Oligonucleotides for RT-qPCR	See Table S7	N/A

**Software and algorithms**		

Imaris (version 9.3)	http://www.bitplane.com/imaris/imaris	RRID: SCR_007370
ImageJ	https://imagej.net/	RRID: SCR_003070
R (version 4.1.0)	https://www.r-project.org/	RRID: SCR_001905
ggplot2 (version 3.0.0)	Wickham (2016)	RRID: SCR_014601
lme4 (version 1.1-17)	Bates et al. (2015)	RRID: SCR_015654
Python (Version 3.7)	https://www.python.org/	RRID: SCR_008394
MatPlotLib (Version 3.4.1)	https://matplotlib.org	RRID: SCR_008624
statsmodels (Version 0.13.2)	http://www.statsmodels.org/	RRID: SCR_016074
networkx (Version 2.8.2)	https://networkx.org/	RRID: SCR_016864
Cytoscape (Version 3.8.2)	https://cytoscape.org/	RRID: SCR_003032

### Resource availability

#### Lead contact

Further information and requests for resources and reagents should be directed to and will be fulfilled by the lead contact, Sandra Siegert (ssiegert@ist.ac.at).

#### Materials availability

This study did not generate new unique reagents.

### Experimental model and subject details

#### Ethical approval

The ISTA Ethics Officer and Ethics Committee approved the use of human induced pluripotent stem cells (hiPSC). The use of human brain samples was approved by the Ethics Committee of the Medical University Vienna.

#### Cell lines

This study used two human induced pluripotent stem cell lines: SC 102A-1 GVO-SBI Human Fibroblast-derived (feeder-free) iPSC cell line (BioCat; male; hPSCreg.eu: SBLi006-A; in this study referred to SC102A). NCRM-5 (aka NL-5; human umbilical cord blood CD34^+^ cells derived; RUCDR Infinite Biologicals, Cell line ID: CR0000005, NHCDR ID: ND5003; male; hPSCreg.eu: CRMi001-A; in this study referred to CR05). For more details, see (Table S1).

#### Primary human tissue samples for confirming antibody specificity

Human brain sample was explanted from the temporal cortex (T1) of a 35-year-old, female patient diagnosed with temporal lobe epilepsy. Immediately after the surgical explant, the samples were transferred into saline solution (0.9% (v/v) NaCl (Braun Cat#3570160) in H_2_O). The tissue was immersed in 4% (w/v) PFA 7 minutes after the explant and post-fixed on an orbital shaker at 4°C overnight.

### Method details

#### Cell culture

##### Matrigel-coating

Matrigel (Corning® Matrigel® hESC-Qualified Matrix, ∗LDEV-Free, (Corning, Cat#354277) was used according to the manufacturer instructions with the following modifications: Matrigel aliquots were dissolved in ice-cold X-Vivo 10 chemically defined, serum-free hematopoietic cell medium (Lonza, Cat#BE04-380Q) prior coating the plates. 6-cm dishes (VWR, Cat#734-0007) were coated for unguided retinal organoid or BMP4-guided differentiation protocols and 2-well chambered coverslips (Ibidi, Cat#80286) for 2.5D culture.

##### Maintenance of human induced pluripotent stem cells

hiPSCs were cultured at 37°C and 5% CO_2_ in a humidified incubator (BINDER C150) in mTeSR1 medium (STEMCELL Technologies, Cat#85850) on Matrigel coated 6-well plates (Corning, Cat#3516). Cells were passaged in small aggregates every 3-4 days and were dissociated before reaching 80% confluency using EDTA dissociation buffer (0.5M EDTA (ethylenediaminetetraacetic acid, K.D. Biomedical, Cat#RGF 3130), 0.9 g (w/v) NaCl (Sigma, Cat#5886) in PBS (phosphate buffered saline, calcium/magnesium-free, Invitrogen, Cat#14190), sterile filtered, stored at 4°C) according to (Chen, 2012). Cells were tested on regular basis for mycoplasma using MycoAlert Mycoplasma Detection Kit (Lonza, Cat#LT07-518). For iPSC differentiation, two wells of a 6-well plate were used for SC102A and four wells for CR05 as starting material.

##### Unguided (retinal organoid differentiation) protocol

3D-retinal organoids were generated similar to (Zhong et al., 2014): On day 0 of differentiation, iPSC colonies were dissociated into evenly sized aggregates using a cell-passaging tool (Thermo Fisher Scientific, Cat#23181-010). After mechanical scraping, floating aggregates were transferred with a 1250μl wide orifice pipette (VWR, Cat#613-0737) onto one 10 cm Petri dish (Sarstedt, Cat#82.1473), and cultured in mTeSR1 medium supplemented with 10 μM blebbistatin (Sigma, Cat#B0560-5MG). On day 1, 2 and 3, the medium was gradually replaced with ¼, ½, and 1, respectively, of NIM (neural induction medium: DMEM/F12 (Gibco, Cat#31331- 028), 1x N2 supplement (Gibco, Cat#17502-48), 1% (v/v) NEAA Solution (Sigma, Cat#M7145), 2 μg/ml heparin (Sigma, Cat#H3149-50KU). From day 4 onwards, 10 ml medium was exchanged daily with NIM. On day 8, the floating embryoid bodies (EB) were collected, equally distributed onto 8 Matrigel-coated 6-cm dishes (approximately 20-40 number of EBs/cm^2^) and cultured in 3 mL NIM. From day 16 onwards, NIM was exchanged daily for 3:1-DMEM/F12-medium (3 parts DMEM (Thermo Fisher Scientific, Cat#31966047) and one-part F12 medium (Ham’s F-12 Nutrient Mix, Thermo Fisher Scientific, Cat#31765-027), supplemented with 2% (v/v) B27 without vitamin A (Thermo Fisher Scientific, Cat#121587-10), 1% (v/v) NEAA solution, 1% (v/v) penicillin-streptomycin (Thermo Fisher Scientific, #15140122). On day 28-32, optic-cup structures were manually micro-dissected from the 6-cm plate and transferred into a 3.5-cm Petri dish (Corning, Cat#351008) containing 2.5 mL 3:1-DMEM/F12-medium. 3:1-DMEM/F12-medium was exchanged twice per week. From day 42 onwards, 3:1-DMEM/F12-medium was supplemented with 10% (v/v) heat-inactivated FBS (Thermo Fisher Scientific, Cat#10270-106) and 100 μM taurine (Sigma, Cat#T0625- 25G). At week 10, the 3:1-DMEM/F12-medium was supplemented with 10 μM retinoic acid (Sigma, Cat#R2625), and the medium was daily exchanged. At week 14, B27 supplement in the 3:1-DMEM/F12-medium was replaced with 1x N2 supplement, 10% (v/v) heat-inactivated FBS, 100 μM taurine and the retinoic acid concentration was reduced to 5 μM.

##### Unguided (retinal organoid differentiation) protocol – Maintenance beyond day 28-32 in 2.5D culture

The differentiation protocol is identical to the “retinal organoid differentiation” section with the following modifications: On day 8, EBs within a volume of 1.5 mL were transferred on Matrigel-coated 2-well chambered coverslip. After the change to the 3:1-DMEM/F12-medium on day 16, 2.5D cultures were exclusively maintained in this media with daily media changes without any additional supplements that are typically added at later differentiation time points in the “retinal organoid differentiation”.

##### BMP4-guided cystic compartment and microglial-like cell differentiation protocol

The differentiation protocol is identical to the “Unguided (retinal organoid differentiation) protocol – Maintenance beyond day 28-32 in 2.5D culture” section with the following differences: On day 1, 12.5 ng/mL (final concentration) of recombinant human BMP4 (Peprotech, Cat#120-05) was added as a single shot. From D8 onwards, medium was exchanged twice per week.

##### Harvesting microglia-like cells after BMP4 application

From D40 onwards, microglia-like cells released into the supernatant were harvested. For this, the supernatant was collected and centrifuged (VWR, Mega Star 3.0R) at 200g for 4 minutes. Cells were resuspended in 3:1-DMEM/F12-medium, and transferred into 8-well chambers (IBIDI, Cat#80826) for immunostaining.

##### Harvesting cystic structure after BMP4 application

At D18, D21, D28, D35 floating cystic structures were transferred into a new 3.5 cm petri dish using a 1250μL wide orifice pipette tip and cultured in 2 mL 3:1-DMEM/F12-medium in parallel to not-transferred cysts, which were further cultured in the original differentiation dish until D45. The medium was exchanged twice per week until D45 when all time points were fixed as described in the results section.

##### Culturing 3D-retinal organoids within BMP4-guided cystic compartments

At D118, eight retinal organoids were transferred into a dish containing BMP4-guided cystic compartment and microglia-like cells and cultured for 10 days. The medium was exchanged to 3:1-DMEM/F12-medium supplemented with 1x N2 Supplement, 10% (v/v) heat-inactivated FBS, and 100 μM taurine. 3 mL medium was exchanged twice per week and 5 μM retinoic acid was added daily.

##### Supplementing 3D-retinal organoids with microglia-like cells

At D118, eight retinal organoids were transferred into a 24 well plate. Microglia-like cells were harvested as described “Harvesting microglia-like cells after BMP4 application” from two 6 cm dishes and added to the organoids once. The medium was exchanged to 3:1-DMEM/F12-medium supplemented with 1x N2 Supplement, 10% (v/v) heat-inactivated FBS, and 100 μM taurine. 3D-retinal organoids and microglia-like cells were cultured for 10 days. 2 mL medium was exchanged twice per week and 5 μM retinoic acid was added daily.

#### Functional assays for microglia-like cells

##### Phagocytosis bead assay

Microglia-like cells were generated with the BMP4-guided protocol, harvested between D40 and D50 from the culture supernatant of a 6-cm dish, and transferred into one well of an 8-well chamber. Microglia-like cells were cultured in 3:1-DMEM/F12-medium for 24 h. Before imaging, cells were washed once with 1x DPBS (Thermo Fisher Scientific, Cat#14190-250) and stained with Tomato-Lectin (Szabo-Scandic, Cat#VECDL-1174, 1:1000 in 1x DPBS) for 20 minutes at 37°C. Then, cells were washed with 1x DBPS, and L15 medium (Thermo Fisher Scientific, Cat#21083027) was added. Images were acquired with a Zeiss LSM880 inverted microscope and a Plan-Apochromat 20x/NA 0.8 Air objective in a temperature-controlled chamber (37°C). Z-stacked images of the 488 and 568 channel were captured simultaneously every minute. After 20 minutes baseline recording, sonicated pH-sensitive fluorescent beads (Thermo Fisher Scientific, Cat#P35361, 1:40) diluted in L15 medium were added, and cells were imaged for the following 60 minutes. For analysis, surface renderings were generated of z-stacks of the entire image using the surface rendering function in Imaris 9.3 with the surface detail setting of 0.2 μm. Next, the intensity mean of the 568 channel was determined within the microglia-like cell created surfaces.

##### Real-time quantitative PCR (RT-qPCR) for inflammatory markers

Microglia-like cells were generated with the BMP4-guided protocol, harvested between D40 and D50 from the supernatant of eight 6-cm dishes, and seeded into a 24-well plate to reach a confluency of 60-80% per well. Cells were incubated overnight at 37°C, 5% CO_2_. Microglia-like cells were treated with human IFN-γ (Sigma-Aldrich, Cat#SRP3058-100UG) and IL1-β (Thermo Scientific, Cat#RIL1BI) or both with a final concentration of 10ng/mL of each cytokine per well, with LPS (Sigma-Aldrich, Cat#L5886-10MG) with a final concentration of 100 ng/mL per well and with poly I:C (Tocris, Cat#4287) with a final concentration of 50 μg/mL per well. Untreated controls received 3:1-DMEM/F12-medium. After 6h of incubation (37°C, 5% CO_2_), RNA was isolated with innuPREP RNA Mini Kit 2.0 (Analytik-Jena, Cat#845-KS-2040050) as described in the manufacturer’s instructions. cDNA synthesis was performed with LunaScript RT SuperMix Kit (New England Biolabs, Cat#E3010L) with a total RNA amount of 200-800ng (same amount for each condition within experimental repetition) and stored at -20°C. Gene expression analysis was performed with Luna Universal qPCR Master Mix (New England BioLabs; Cat#M3003L) in 384 well plates (Bio-Rad; Cat#HSR4805) on a Roche Lightcycler 480 using the device’s “Second Derivative Maximum Method”. Total reaction volume was 10μl containing 1μl of 1:10 diluted cDNA. The final concentration for each primer was 0.25μM (Table S7). Cycle conditions were as follows: Initial denaturation (60 second; 95°C), 40 cycles of denaturation (15 seconds; 95°C) and annealing/extension (30 seconds; 60°C). PCR reactions were run in triplicates from which a mean Cq value was calculated. Mean Cq values were normalized to the geometric mean of four reference genes (GAPDH, ACTB, OAZ1, RPL27) measured within the same sample to obtain dCq. dCq values were normalized to control condition (untreated cells) within each experimental repetition to calculate ddCq values. For data visualization, ddCq values from log2-scale were used to describe fold changes between the treated and untreated group.

##### Real-time quantitative PCR (RT-qPCR) for microglia markers

Microglia-like cells were harvested between D40 and D50 and cDNA synthesis was performed as described under “Real-time quantitative PCR (RT-qPCR) for inflammatory markers” with following adaptations: RNA was isolated 24h after microglia-like cells were seeded. PCR was performed using primers listed in Table S7.

##### Ca^2+^ imaging

Microglia-like cells were generated with the BMP4-guided protocol, harvested between D40 and D50 from the supernatant of two 6-cm dishes, and were transferred into two wells of an 8-well chamber. Microglia-like cells were cultured in 3:1-DMEM/F12-medium for 24 h. Cells were labeled with Fluo-4 (Invitrogen, Cat#F10471; reconstituted at 1X in supplied buffer) for 30 minutes at 37°C and 5% CO_2_. Afterwards, cells were further incubated at room temperature (light-protected) and atmospheric CO_2_ for another 30 minutes. Labeling solution was aspirated and L15 medium was added. Single-plane 16-bit images were acquired with a frame rate of 500 ms for a total duration of 360 seconds with LSM880 inverted microscope and a 20x air objective. After 180 seconds of baseline recording, 1 mM ATP (final concentration) or L15 medium was applied. Fluorescent intensity levels of Ca^2+^ events occurring in IBA1^+^-cells were recorded for the following 180 seconds minutes. Images were processed in Fiji 1.51 by applying a Gaussian filter with a sigma of 1.0. Regions of interest (ROIs) were drawn on the center of individual cells. For each frame, intensity was measured. The data was visualized by normalizing the intensity of each cell to its average intensity throughout the entire 360 seconds recording. For Figure S6C, Ca^2+^ events were automatically detected with the software PeakCaller (Artimovich et al., 2017) using following parameters: required rise = 20% absolute; max. lookback = 700 pts; required fall = 30% absolute; max. lookahead = 700 pts; trend control = exponential moving average (2-sided); trend smoothness = 100; interpolate across closed shutters = true. The output was additionally filtered in R by including only peaks with a height greater than 0.15 and a FWHM greater than 5, to remove erroneously detected Ca^2+^ events.

#### Histology

##### Histology - human brain samples

After PFA fixation, the samples were washed with PBS at least for 15 minutes three times. The samples were embedded in 3% (w/v) agarose (Sigma, Cat#A9539) and sliced with a vibratome (Leica VT 1200) at a thickness of 100 μm. The vibratome slices were then cryoprotected with 30% (w/v) sucrose (Sigma, Cat#84097, sterile filtered) until they sunk in the solution. The samples were stored at -80°C until further use.

##### Fixation of 3D-retinal organoids/cystic structures (=aggregates)

Aggregates were fixed in 4% (w/v) PFA in PBS for 20 minutes at room temperature, then washed three times with PBS at room temperature and cryopreserved in 30% (w/v) sucrose in PBS overnight at 4°C.

##### Cryostat sectioning

Cryopreserved aggregates were transferred to a cryomold (PolyScience, Cat#18985) using a 1250μL wide orifice pipette tip and embedded in Tissue-Tek O.C.T. compound (TTEK, A. Hartenstein) on dry ice. Samples were stored at -80°C until further use. Cryosections (20-30 μm) of aggregates were generated using a cryostat (MICROM, NX70 CRYOSTAR, Thermo Scientific). Sections were mounted onto Superfrost Plus glass slides (Lactan, Cat#H867.1), dried at room temperature overnight and stored at -80°C until further use. For immunostaining, slides were dried for 1 h at room temperature. Sections on glass slices were encircled with an engraving, hydrophobic pen (Sigma-Aldrich, Cat#Z225568).

##### Immunostaining of cryostat sections

Cryostat sections were incubated in “blocking solution” containing 1% (w/v) bovine serum albumin (Sigma, Cat#A9418), 5% (v/v) Triton X-100 (Sigma, Cat#T8787), 0.5% (w/v) sodium azide (VWR, Cat#786-299), and 10% (v/v) serum (either goat, Millipore, Cat#S26, or donkey, Millipore, Cat#S30) for two hours in a humidified chamber protected from light at room temperature. Afterwards, the samples were immunostained with primary antibodies diluted in antibody solution containing 1% (w/v) bovine serum albumin, 5% (v/v) triton X-100, 0.5% (v/v) sodium azide, 3% (v/v) goat or donkey serum, and incubated overnight in a humidified chamber at room temperature. For the list of primary antibodies and their dilutions see key resources table.

The sections were washed three times with PBS and incubated in a light-protected humidified chamber for 2 hours at room temperature, with the secondary antibodies diluted in antibody solution. The secondary antibodies raised in goat or donkey were purchased from Thermo Fisher Scientific (Alexa Fluor 488, Alexa Fluor 568, Alexa Fluor 647, 1:2000). The sections were washed three times with PBS. The nuclei were labeled with Hoechst 33342 (Thermo Fisher Scientific, Cat#H3570, 1:5000 diluted in PBS) for 8 minutes, and after a final two times PBS wash embedded using an antifade solution [10% (v/v) mowiol (Sigma, Cat#81381), 26% (v/v) glycerol (Sigma, Cat#G7757), 0.2M tris buffer pH 8, 2.5% (w/v) Dabco (Sigma, Cat#D27802)] with microscope cover glass slips (Menzel-Glaser, Cat#0). Samples were stored at 4°C until imaging.

##### Immunostaining for human brain slices

The staining was performed as described under “Immunostaining of cryostat sections” with following adaptations: Floating brain slices were stained in a 24-well plate and the primary antibody was incubated for 48 hours on a shaker. After immunostaining, the slices were mounted on glass microscope slides (Assistant, Cat#42406020) and embedded with antifade solution.

##### Immunostaining for whole mount aggregates

The staining was performed as described under “Immunostaining of cryostat sections” with the following adaptations: The primary antibody was incubated for 48 hours on a shaker at room temperature, and washed at least for two hours.

##### Immunostaining for whole mount organoids

The staining was performed as described under “Immunostaining of cryostat sections” with the following adaptations: Organoids were incubated in blocking solution for 2 days on a shaker at 4°C. The primary antibody concentration was doubled and organoids were incubated for 10 days on a shaker at 4°C, and washed three times in PBS at least for one day. Then the organoids were incubated with secondary antibodies (1:500) and Hoechst (1:1000) diluted in antibody solution simultaneously for 3 days on a shaker at 4°C. After washing the organoids three times in PBS for one day, organoids were mounted with low gelling agarose followed by a glycerol gradient as described in “Immunostaining for whole mount aggregates”.

##### Mounting of whole mount aggregates

For whole mount aggregates, the tissue was mounted on 8-well chambers using 3% (w/v) low gelling temperature agarose (Sigma-Aldrich, Cat#A9414-25G). Then a glycerol gradient was performed starting with 50% (v/v) glycerol in H_2_O followed by 75% (v/v) glycerol in H_2_O. Afterwards, the whole mount aggregate was imaged.

#### Imaging and analysis

##### Brightfield

Differentiation was monitored with a bright-field microscope (Olympus CKX41) with 5x, 10x and 20x objectives (Olympus) and a lens marker (Nikon), and an EVOS microscope (Thermo Fisher Scientific) with 2x, 4x, 10x, 20x, 40x objectives (Thermo Fisher Scientific).

##### Confocal microscopy

Images were acquired with a Zeiss LSM880 Airyscan upright or inverted or with a Zeiss LSM800 upright. Ibidi plates were exclusively imaged using an inverted microscope. For overview images Plan-Apochromat 10x air objective NA 0.45 (WD=2.1mm) or Plan-Apochromat 20x Air objective NA 0.8 were used and tile-scan z-stacks were acquired. For detailed images Plan-Apochromat 40x oil immersion objective NA 1.3 was used.

##### Image analysis

Confocal images were converted to .ims files using the Imaris converter and imported to Imaris 9.3 (Bitplane Imaris 3/4D Image Visualization and Analysis Software).

*Surface rendering* were generated using the surface rendering module with the surface detail set to 0.2 μm.

##### Determining the volume of organoids

The Hoechst channel was processed using the normalize layer function of Imaris. Then, a surface rendering was performed and the total volume of the Hoechst channel was determined.

##### Determining the number of IBA1^+^-cells

The spot function of Imaris was used to analyze the number of IBA1^+^-cells. The estimated XY diameter was set to 15 μm.

##### Quantification of types of aggregates

Bright field images of aggregates were acquired and then classified into the four types as outlined in Figure 2A (retinal cup only, retinal cup with cerebral compartment, retinal cup with cystic compartment, cyst only). Then the percent ratio of each of the four types was determined.

##### Graphics

All graphics were generated using R (version 4.1.0). Excel files were loaded into R via the xlsx package (version 0.6.1) (Dragulescu and Cole, 2014). Plots were made using ggplot2 (version 3.0.0) (Wickham, 2016). Linear regression was performed using the lme4 package (version 1.1-17) (Bates et al., 2015).

#### Mass spectrometry

10 cystic structures were harvested at D28 and D45, washed once in DPBS (Thermo Fisher Scientific, # 14190-250) and snap frozen in liquid nitrogen. Samples were stored at -80°C until further analysis. For Liquid chromatography - mass spectrometry (LCMS) analysis, pelleted cystic structures were denatured, reduced, alkylated with iodoacetamide and trypsin-digested into peptides using a commercial in-Stage Tips kit (P.O.00001, Preomics), following exactly the manufacturer’s instructions. Cleaned-up, reconstituted peptides were then analyzed by Liquid chromatography – tandem mass spectrometry (LC-MS/MS) on an Ultimate High-performance liquid chromatography (HPLC) (ThermoFisher Scientific) coupled to a Q-Exactive HF (ThermoFisher Scientific). Each sample was concentrated over an Acclaim PepMap C18 pre-column (5 μm particle size, 0.3 mm ID x 5 mm length, ThermoFisher Scientific) then bound to a 50 cm EasySpray C18 analytical column (2 μm particle size, 75 μm ID x 500 mm length, ThermoFisher Scientific) and eluted over the following 180 min gradient: solvent A, water + 0.1% formic acid; solvent B, 80% acetonitrile in water + 0.08% formic acid; constant 300 nL/min flow; B percentage: start, 2%; 155 min, 31%; 180 min, 44%. Mass spectra were acquired in positive mode with a Data Dependent Acquisition method: FWHM 20s, lock mass 445.12003 m/z; MS1: profile mode, 120,000 resolving power, AGC target 3e6, 50 ms maximum IT, 380 to 1,500 m/z; MS2: top 20, centroid mode, 1.4 m/z isolation window (no offset), 1 microscan, 15,000 resolving power, AGC target 1e5 (minimum 1e3), 20 ms maximum IT, 200 to 2,000 m/z scan range, NCE 28, excluding charges 1 and 8 or higher, 60s dynamic exclusion.

Raw files were searched in MaxQuant 1.6.5.0 against the reference *Homo sapiens* proteome downloaded from UniProtKB. Fixed cysteine modification was set to Carbamidomethyl. Variable modifications were Oxidation (M), Acetyl (Protein N-term), Deamidation (NQ), Gln->pyro-Glu and Phospho (STY). Match between runs, dependent peptides and second peptides were active. All FDRs were set to 1%.

##### Tissue enrichment analysis

To determine human tissues that resemble the highly expressed protein profile in the cystic structure, we performed a tissue enrichment analysis, which employs hypergeometric testing. In our case, proteins specific to a given tissue (or *tissue-specific proteins*) were downloaded from the Human Protein Atlas (HPA, http://www.proteinatlas.org) (Uhlén et al., 2015) which has curated the expression profiles of human genes both on the mRNA and protein level in 44 normal human tissue types (corresponding to 62 tissue samples). In particular, tissue-specific proteins were defined as proteins that were highly detected, i.e., a strong immunohistochemical staining intensity in 25-75% of cells as annotated in HPA. Cystic-specific proteins in either D28 or D45, on the other hand, were obtained by taking proteins whose expressions are within the 98^th^ percentile of the protein expression distribution in the mass spectroscopy data. The overlap between the 3D-cyst specific proteins and the tissue-specific proteins were calculated and the hypergeometric test was used to calculate the enrichment of this overlap as

$$P\left(X\geq k\right)=\sum_{i\geq k}\frac{\left(\begin{array}{l} M\\ i \end{array}\right)\left(\begin{array}{l} N-M\\ n-i \end{array}\right)}{\frac{N}{n}}$$

with *n* as the number of 3D-cyst specific proteins from the *N* total number of detected proteins with mass spectrometry, *M* as the number of tissue-specific proteins and *k* is the size of their overlap. The obtained *p*-values were Bonferroni-corrected for multiple comparisons implemented through the multitest function of statsmodels (Seabold and Perktold, 2010).

This strategy has bene also applied in both Angeles-Albores et al., 2016; Jain and Tuteja, 2019 to identify the tissue identity of a gene set from a gene-tissue association library constructed from *C. elegans* genes and from tissue-specific RNAseq data, respectively. A similar analysis was conducted for proteins found in previous mass spectrometry analyses of human iris, ciliary body, RPE/choroid (Zhang et al., 2016a), optic nerve, sclera (Zhang et al., 2016b), retina (Zhang et al., 2015) and meninges (Dunn et al., 2019). Tissue-specific protein profiles were defined as the proteins that are present in the 80th percentile of the protein expression distribution. Tissue-specific proteins that are in at most two tissues were discarded to account for possible non-specific expression. Note that while the tissue enrichment p-values change with the percentile cut-off, the qualitative results remain the same.

##### Heatmap of mesenchymal stem cell markers

The list of markers of epithelial and mesenchymal markers was obtained from (Andrzejewska et al., 2019; Owusu-Akyaw et al., 2019; Scanlon et al., 2013). Protein expression in 3D cysts for these markers was plotted as a heatmap for week 4 and 7. Fold-change was determined by dividing the intensity at D45 with the intensity at D28. Upregulated proteins are those with fold-change greater than or equal to 2.0 while downregulated proteins are those with fold-change less than or equal to 0.5.

### Quantification and statistical analysis

All statistical tests were performed using R. Models were generated by changing the default contrast for unordered variables to “contr.sum” to apply type III ANOVA to the model to evaluate the overall contribution of the response variable. Post-hoc tests were performed via the “dplyr” package (version 1.0.7) (Wickham et al., 2021) and the “multcomp” package (version 1.4-17) and were corrected for multiple testing using the single-step method (Hothorn et al., 2008). Pearson correlation was performed using the “ggpubr” package (version 0.4.0) (Kassambara, 2017). Statistical details of experiments can be found in the figure legends, including the statistical tests used, definition of center and dispersion and precision measures.

#### Overview of applied statistical tests

##### Inflammation assay

A one-sample t-test was performed to compare the stimulated condition with its untreated control (Figure 3H).

##### 3D cyst occupation and isolation

We performed a Pearson correlation test to the correlation between IBA1+-cell density and age of differentiation (Figures 4B and 4D).

##### Co-expression of IBA1 and CD163

We performed two-way ANOVA to examine changes in expression over time by using an interaction of these two predictors. A random effect (cyst ID) was included to account for the dependency of the data which results from repeated counting of the same sections. As a significant effect (p < 0.05) was observed for the interaction we performed a post-hoc analysis for pair-wise comparison using the Tukey-Test and p-values were adjusted using the method set to “BH” (Figure 8C).

##### Integration of IBA1^+^-cells into 3D-retinal organoids

A Shapiro-Wilk test determined that the data was not normally distributed. Therefore, we performed a Wilcoxon-test to test differences between experimental conditions (Figure S7D).

##### Repetition

All experiments were performed by at least two experimentalists independently for both cell lines with exceptions of the mass spectrometry (Figure 5) and (Figures 4A–4D). In total, we performed for the hiPSC lines SC102A 18x and CR05 10x retinal organoid differentiations and for SC102A 10x and CR05 8x BMP4-guided differentiation.

## Data Availability

• The data reported in this paper is available from the lead contact upon request. • The paper does not report original code. • Any additional information required to reanalyze the data reported in this paper is available from the lead contact upon request.

## Supplemental information

Document S1. Figures S1–S8 and Tables S1 and S7

Table S2. Mass spectrometry raw data, related to Figure 5

Table S3. Mass spectrometry for tissue enrichment, related to Figure 5B-C

Table S4. Tissue enrichment analysis enhanced tissues, related to Figure 5B

Table S5. Eye tissue enrichment analysis, related to Figure 5C

Table S6. Mesenchymal and epithelial marker, related to Figure 5D-E
