# Supplementary material for: A systematic characterization of microglia-like cell occurrence during retinal organoid differentiation
Source: iScience. 2022 Jun 11;25(7):104580. doi: 10.1016/j.isci.2022.104580 (PMC9250027; doi:10.1016/j.isci.2022.104580)
Supplement: Document S1. Figures S1–S8 and Tables S1 and S7 [file mmc1.pdf]

iScience, Volume *25*

Supplemental information

A systematic characterization of microglia-like cell occurrence during retinal organoid differentiation

Katarina Bartalska, Verena Hübschmann, Medina Korkut-Demirbaş, Ryan John A. Cubero, Alessandro Venturino, Karl Rössler, Thomas Czech, and Sandra Siegert

Figure S1 (related to Figure 1)

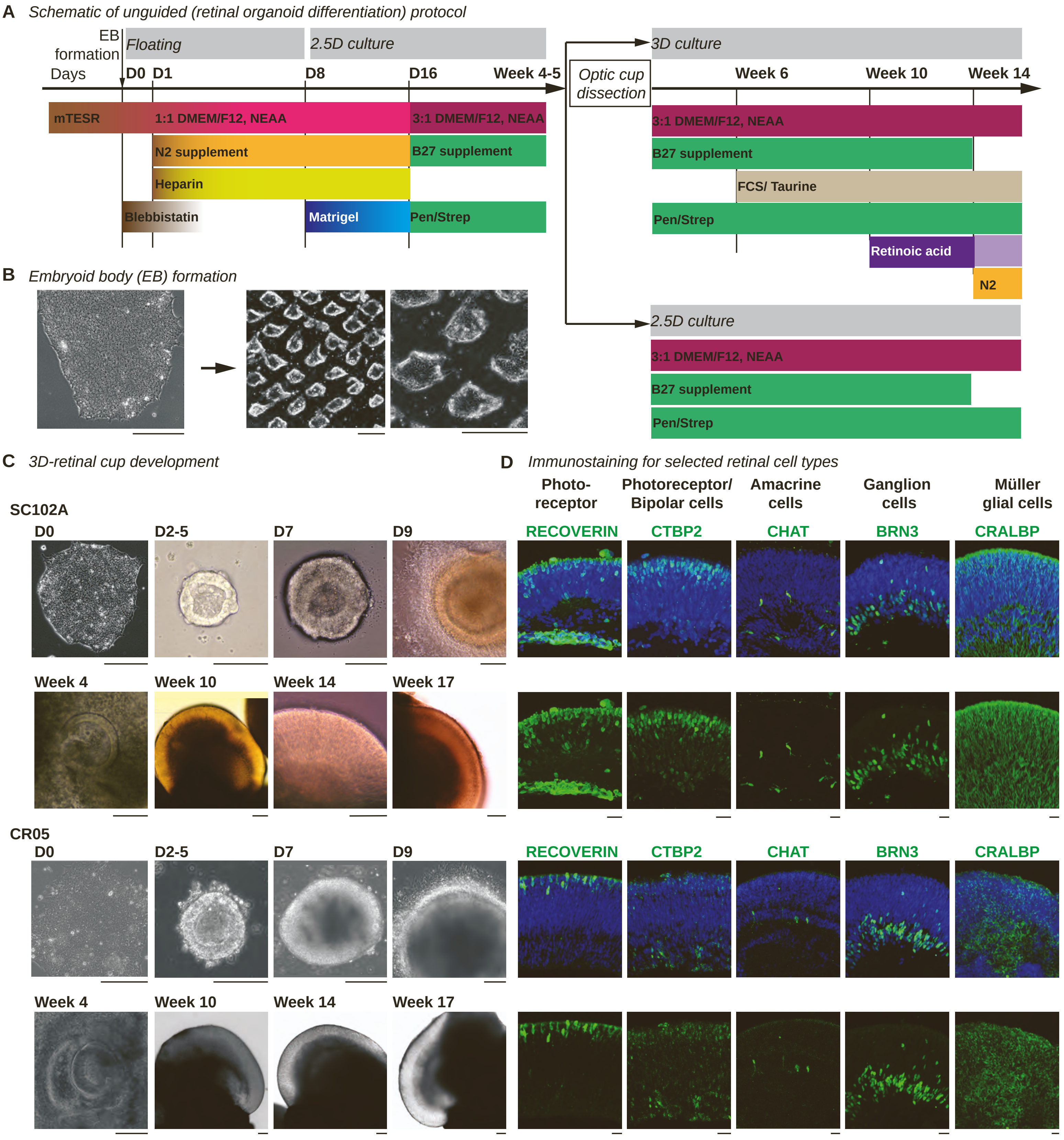

Figure S1. Differentiation of hiPSC lines SC102A and CR05 into 3D-retinal organoids (related to Figure 1).

A-**B**, Schematic of unguided (retinal organoid differentiation) protocol for 2.5D and 3D culture. After reaching 80% confluency, human induced pluripotent stem cells (hiPSC) were cut into evenly sized aggregates to form embryoid bodies (EBs) (**B**, Scale bar: 100 µm). EBs were cultured in suspension and were seeded on D8 on Matrigel-coated plates. At D30-32, optic-cup structures were manually micro-dissected and cultured in suspension. D, days after induced differentiation. FCS, *fetal* calf serum. NEAA, Non-Essential Amino Acid. Pen/Strep, penicillin and streptomycin. **C**, Brightfield images at selected days and weeks after induced differentiation (D) for SC102A (top) and CR05 (bottom). Scale bar: 100 µm. **D**, Immunostaining of cryostat sections for selected retinal cell type marker (green) and the nuclear dye Hoechst (blue) with focus on the retinal-cup for SC102A (top) and CR05 (bottom) between week 18-20 with exception of BRN3 for SC102A at week 9. CTBP2, C-Terminal-Binding Protein 2. CHAT, choline acetyltransferase. BRN3, brain-specific Homeobox/POU Domain Protein 3. CRALBP, cellular retinaldehyde-binding protein. Scale bar: 20 µm.

Figure S2 (related to Figures 1, 2, 4, 6-8, S3-S8)

Validation of antibodies in human temporal lobe brain tissue

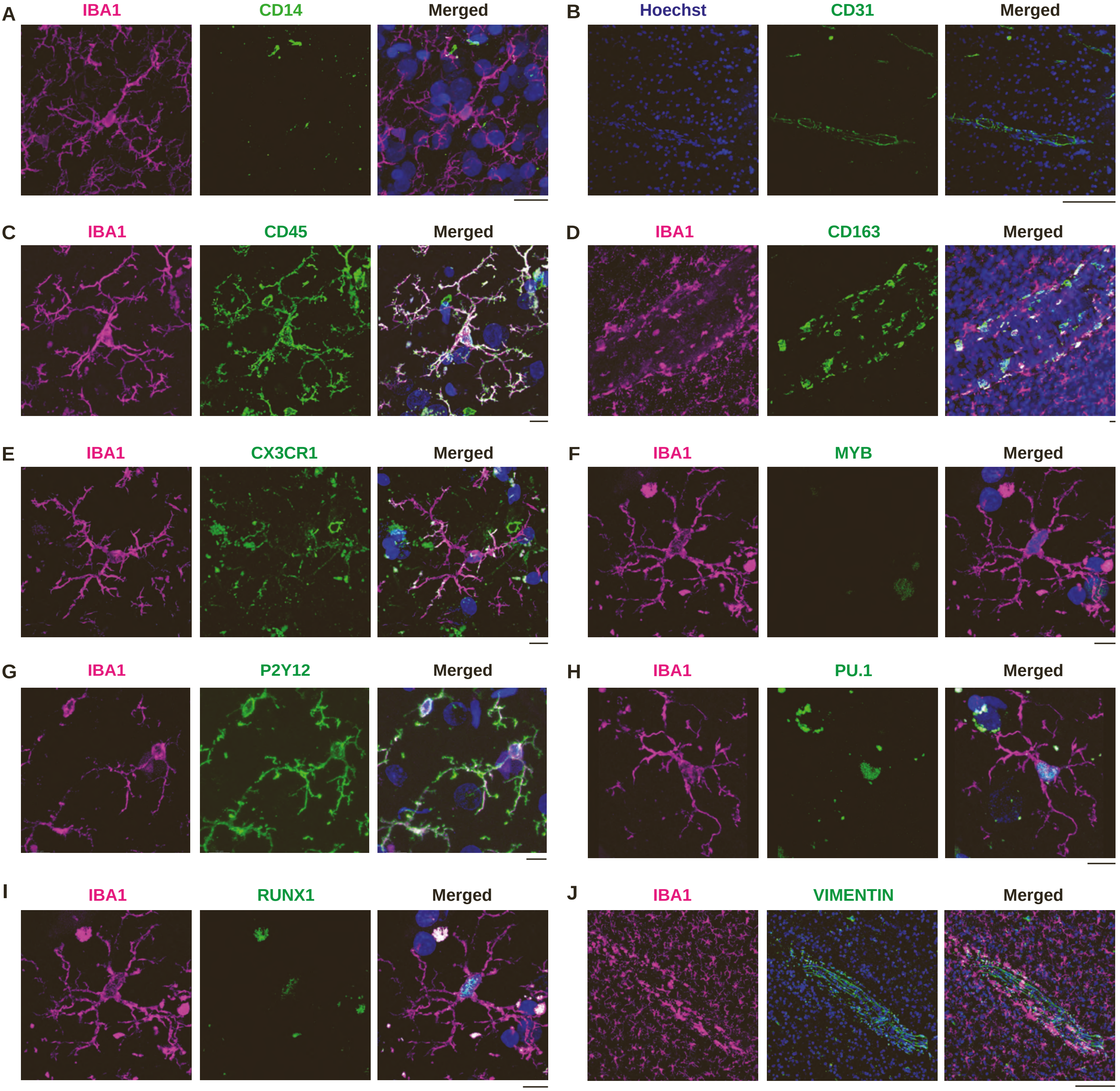

Figure S2. Confirmation of antibody specificity in human brain tissue (related to Figures 1, 2, 4, 6-8, S3-8).

Vibratome sections of adult human temporal lobe immunostained for IBA1 (ionized calcium-binding adapter molecule 1, magenta), nuclei dye Hoechst (blue), and antibodies used throughout this study (green): **A**, CD14, cluster of differentiation 14/ monocyte differentiation antigen CD14. **B**, CD31, platelet endothelial cell adhesion molecule (PECAM-1). **C**, CD45, cluster of differentiation 45/ protein tyrosine phosphatase receptor. **D**, CD163, cluster of differentiation 163/ scavenger receptor cysteine-rich type 1 protein M130. **E**, CX3CR1, Chemokine (C-X3-C) Receptor 1. **F**, MYB, MYB Proto-Oncogene. **G**, P2Y12, Purinergic receptor P2Y G-protein-coupled 12. **H**, PU.1, Hematopoietic transcription factor PU.1. **I**, RUNX1, Runt-related transcription factor 1. **J**, VIMENTIN. Scale bar: 20 µm.

Figure S3 (related to Figure 1)

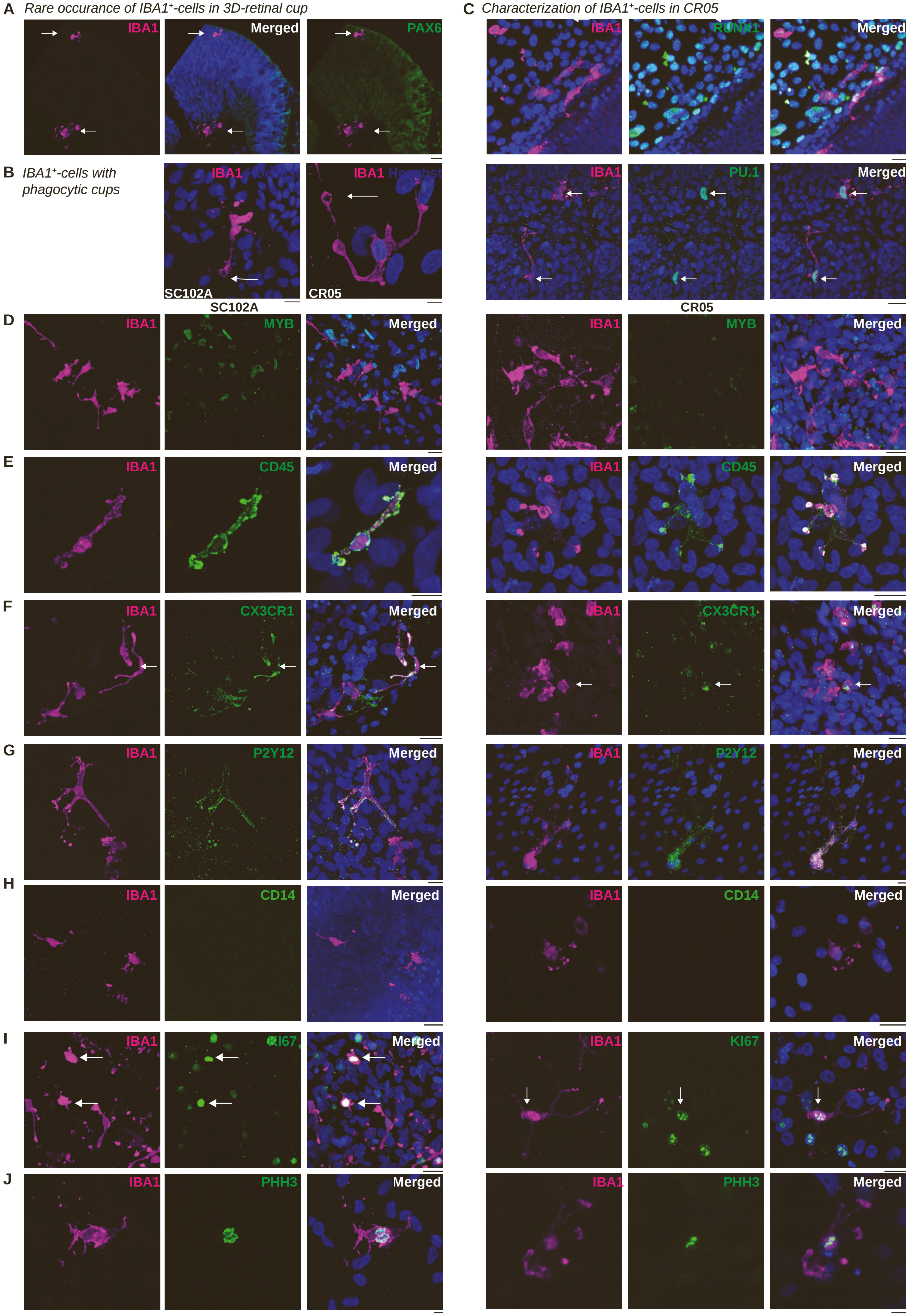

Figure S3. Characterization of IBA1$^+$-cells (related to Figure 1).

Immunostaining for IBA1 (ionized calcium-binding adapter molecule 1, magenta) and the nuclei dye Hoechst (blue). **A**, Cryostat section of 3D-retinal organoids differentiated from the unguided protocol from SC102A at week 9 with focus on retinal cup. White arrow, examples of overlap. **B**-**J**, Immunostaining in 2.5D culture. Scale bar: 20 µm. **B**, Examples of IBA1$^+$-cell forming phagocytic cups (arrow) in aggregates from SC102A (left) and CR05 (right) at week 8. Scale bar: 10 µm. **C**-**D**, Hematopoietic lineage-specific markers between week 4-5. Immunostaining in green: **C**, RUNX1 (runt-related transcription factor 1), PU.1 (hematopoietic transcription factor PU.1) in CR05. **D**-**I**, SC102A (left) and CR05 (right). **D**, MYB (MYB Proto-Oncogene). **E**-**G**, at week 4-5. **E**, Hematopoietic marker CD45 (cluster of differentiation 45/ protein tyrosine phosphatase receptor). **F**, CX3CR1 (Chemokine (C-X3-C) Receptor 1). **G**, P2Y12 (purinergic receptor P2Y G-protein-coupled 12). **H**, CD14 (cluster of differentiation 14/ monocyte Differentiation Antigen CD14). **I**-**J**, Proliferation marker between week 8-10 in green: KI67 (**i**) PHH3 (phosphohistone H3, **J**). Scale bar: 20 µm, if not otherwise indicated.

Figure S4 (related to Figure 3)

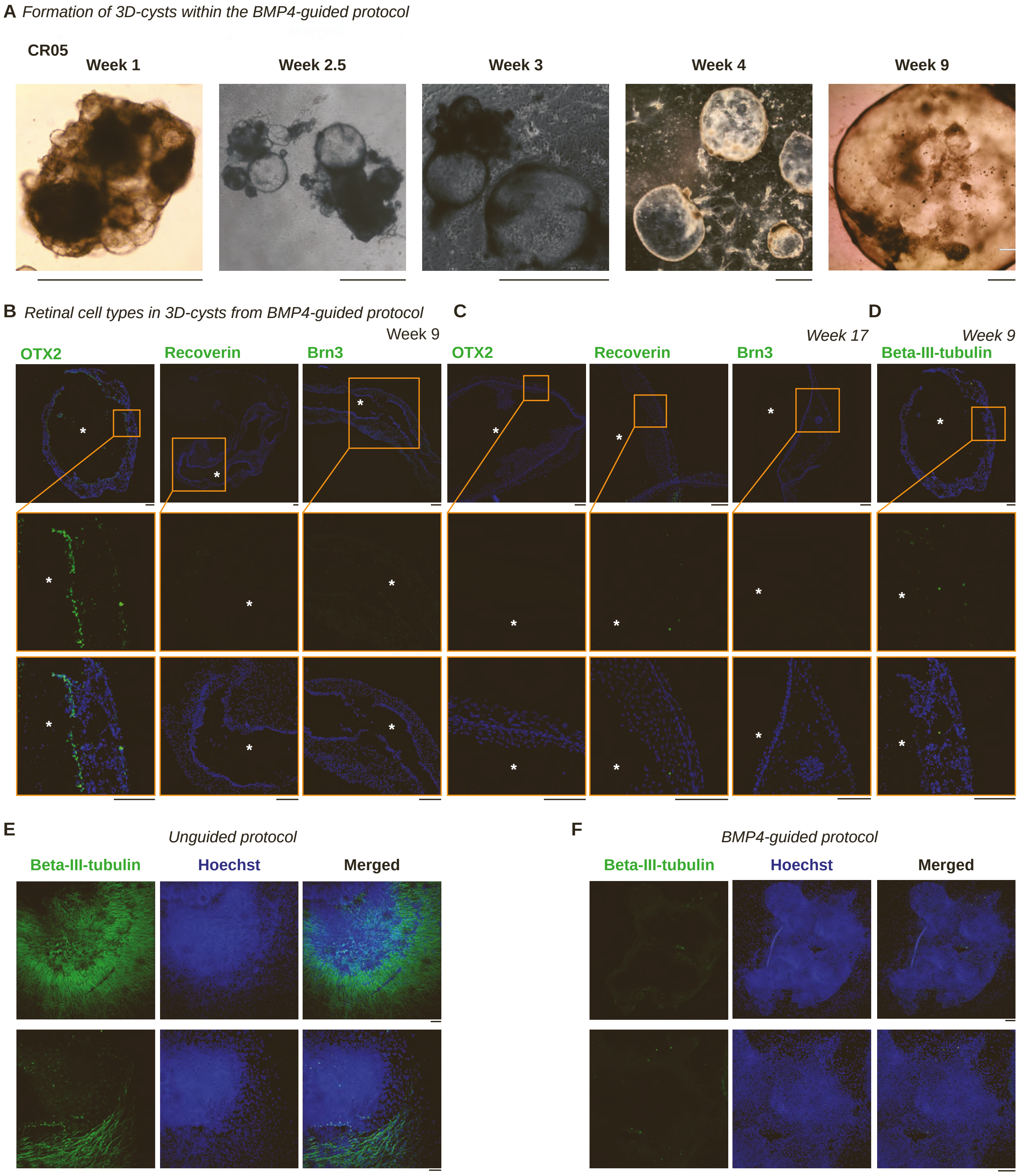

Figure S4. IBA1$^+$-cells occur with BMP4-guided protocol (related to Figure 3).

A, Brightfield images of developing 3D-cysts generated with BMP4-guided protocol for CR05 (week 1-9). Scale bar: 1000 µm. **B-D**, Immunostaining of cryostat sections of 3D-cysts at week 9 (**B**, **D**) and week 17 (**C**) generated with BMP4-guided protocol from SC102A. Green: OTX2 (orthodenticle homeobox 2) for photoreceptors and bipolar cells. Recoverin for photoreceptors. BRN3 (brain-specific Homeobox/POU Domain Protein 3) for ganglion cells (**B**-**C**). Neuronal marker beta-III-tubulin (**d**). Blue: nuclei-dye Hoechst. BRN3, brain-specific Homeobox/POU Domain Protein 3 for ganglion cells. Orange frame, zoom in. *, lumen. Scale bar: 100 µm. **E-F**, Immunostaining of 2.5D culture from SC102A at day 12 from unguided protocol (**E**) and BMP4-guided protocol (**F**) for the neuronal marker beta-III-tubulin (green) and the nuclei dye Hoechst (blue). Scale bar: 100 µm.

Figure S5 (related to Figure 3)

Marker expression of IBA1⁺-cells from BMP4-guided protocol

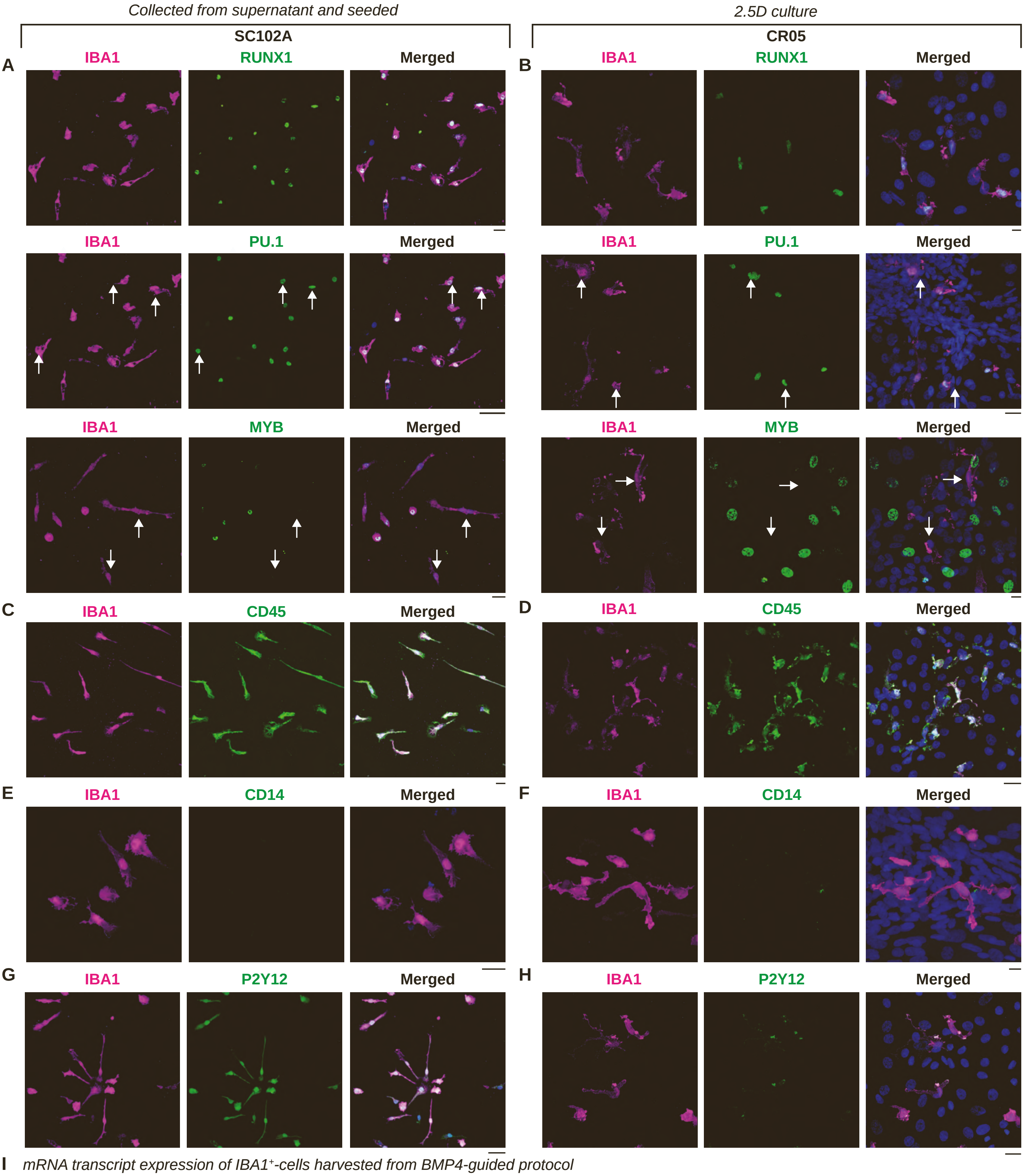

I *mRNA transcript expression of IBA1⁺-cells harvested from BMP4-guided protocol*

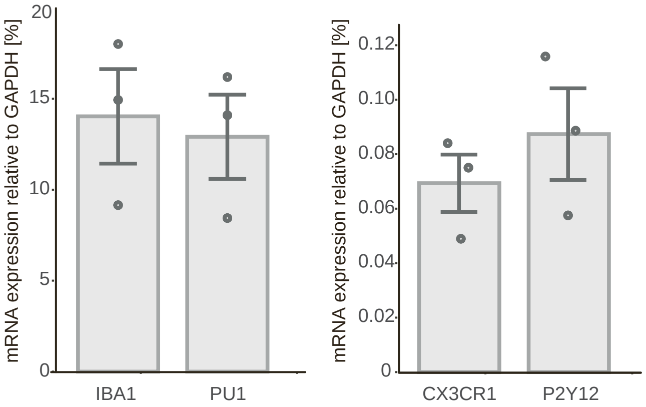

Figure S5. IBA1$^+$-cells occur with BMP4-guided protocol (related to Figure 3).

A-H, Immunostaining for IBA1 (ionized calcium-binding adapter molecule 1, magenta) and nuclei dye Hoechst (blue) obtained from the BMP4-guided protocol either collected and seeded at week 6 from the supernatant of SC102A (**A, C, E, G**), or directly labeled on 2.5D culture plates of CR05 between week 4 and 6 (**B, D, F, H**). In green: Immunostaining for **A-B**, RUNX1 (runt-related transcription factor 1), PU.1 (hematopoietic transcription factor PU.1), and MYB (MYB Proto-Oncogene). White arrow, examples of non-overlap. **C-D**, CD45 (cluster of differentiation 45/ protein tyrosine phosphatase receptor). **E-F**, CD14 (cluster of differentiation 14/ monocyte Differentiation Antigen CD14). **G-H**, P2Y12 (purinergic receptor P2Y G-protein-coupled 12). Scale bar: 20 µm. **I**, mRNA transcript expression of IBA1, PU.1, CX3CR1 and P2Y12 relative to the housekeeping gene GAPDH with mean standard error of IBA1$^+$-cells harvested from the supernatant of BMP4-guided protocol. Each dot represents an independent differentiation.

Figure S6 (related to Figure 3)

A *Phagocytosis assay*

CR05

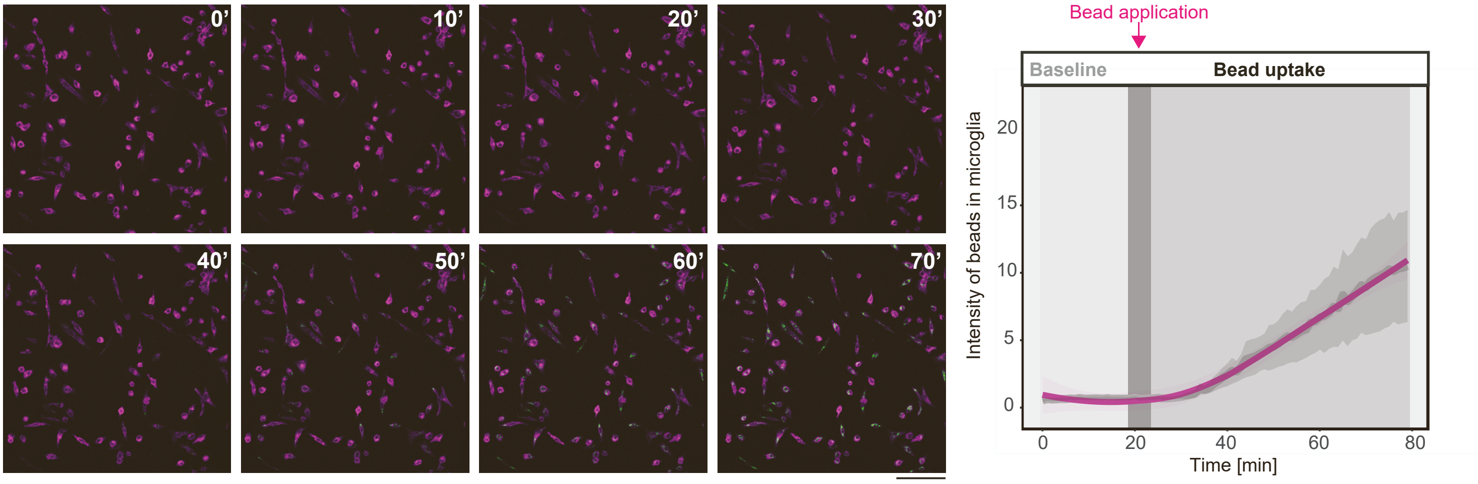

B *Fluorescence intensity of Ca2+ events of IBA1+-cells in medium treated controls*

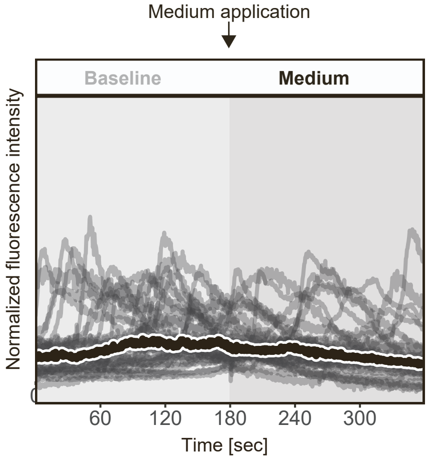

C *Sum of Ca2+ events in IBA1+-cells*

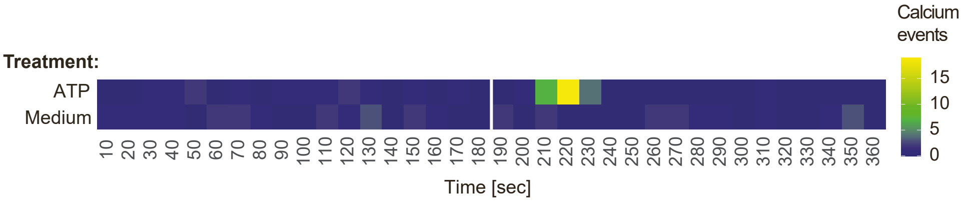

Figure S6. Functional characterization of $IBA1^+$-cells (related to Figure 3).

A, Phagocytosis assay. Left: Consecutive snapshots of live imaged tomato-lectin-labeled microglia-like cells (magenta) collected from the supernatant of the BMP4-guided protocol (CR05) and their uptake of fluorescent beads (green) week 6-7. Scale bar: 100 µm. Mean intensity increase of beads within $IBA1^+$-cells and 95% confidence interval band during 80 minutes of recording of three biological replications. Dark grey bar: bead application after 20 minutes of baseline recording. **B**, Medium treated control of Ca^{2+}-imaging in $IBA1^+$-cells derived from BMP4-guided protocol for SC102A week 6-7. Graph shows Ca^{2+}-dependent fluorescence intensity normalized to the mean intensity of the cells throughout 360 seconds of recording. After 180 seconds of baseline measurement (light grey area), L15 medium was applied, and recording was continued up to 360 seconds (dark grey area). Ca^{2+} events are detected by PeakCaller software. Each curve shows the Ca^{2+} events of an individual cell. Black line: median of 32 cells from three independent differentiations. **C**, Graph shows sum of software-detected Ca^{2+} events from all cells of ATP- and medium treated conditions across time in 10 second-bins. Ca^{2+}-dependent fluorescence is displayed through an intensity-based color code (blue-green-yellow). White vertical line indicates drug application time point.

Figure S7 (related to Figure 7)

A *Adding 3D-retinal organoids without cystic compartment to 2.5D culture of BMP4-guided protocol (Condition 1)*

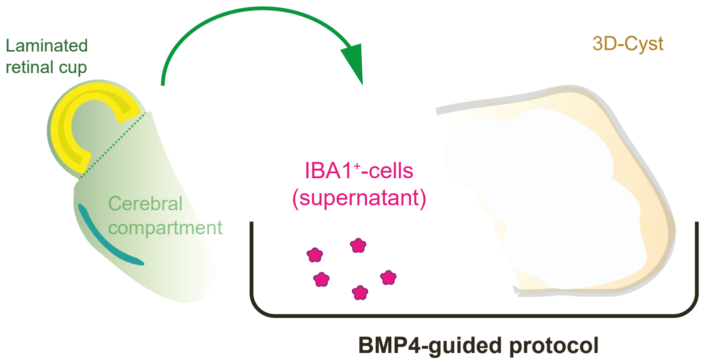

B

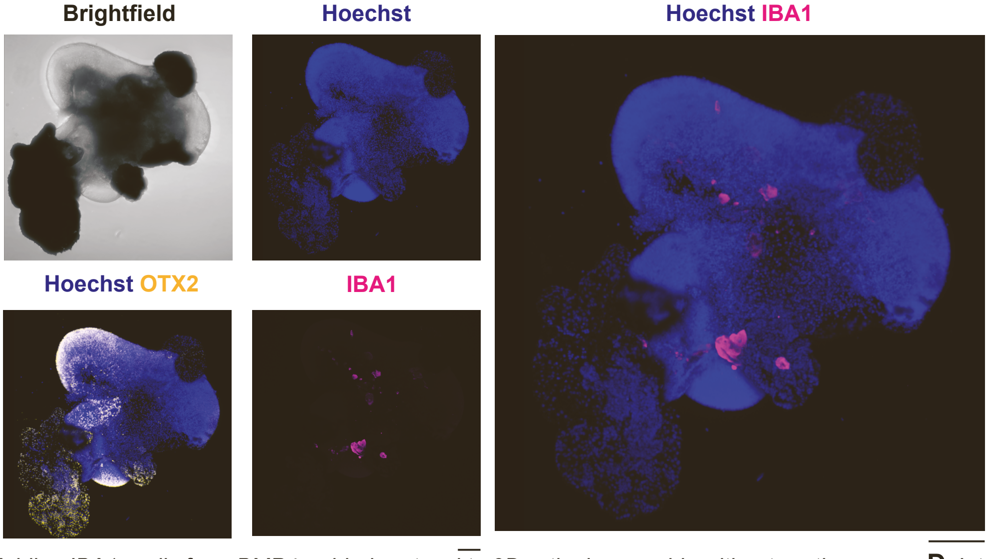

C *Adding IBA1⁺-cells from BMP4-guided protocol to 3D-retinal organoids without cystic compartment (Condition 2)*

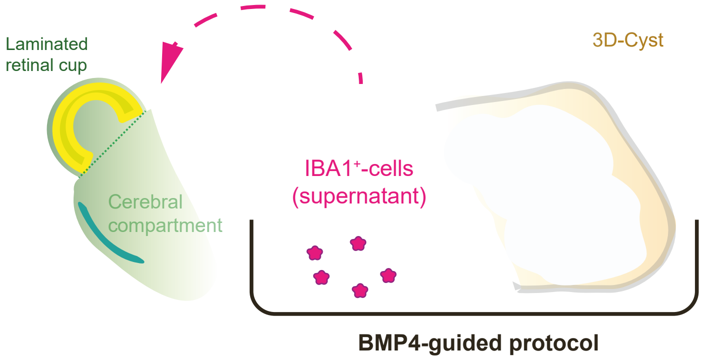

D *Integration of IBA1⁺-cells into 3D-retinal organoids without cystic compartment*

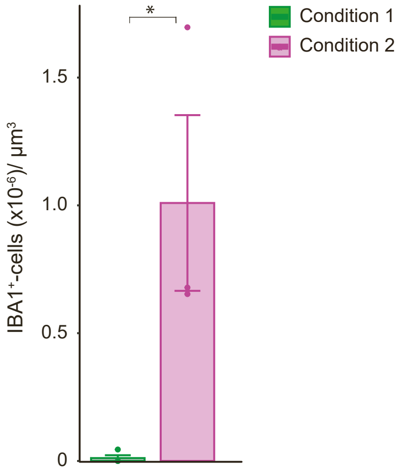

E

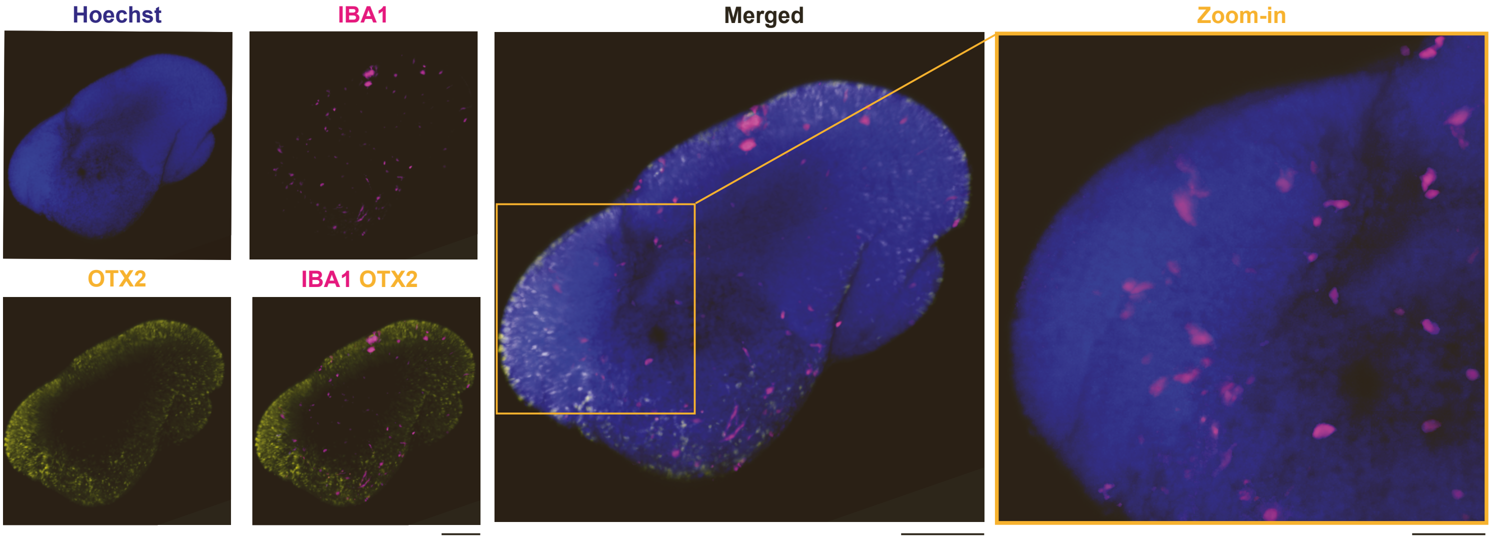

Figure S7. IBA1$^+$-cell preference for 3D-cysts *versus* 3D-retinal organoid (related to Figure 7).

A, Experimental schematic of adding 3D-retinal organoids without cystic compartment generated with unguided protocol to 2.5D culture of BMP4-guided protocol (condition 1). **B**, Whole mount 3D-retinal organoid without cystic compartment from SC102A immunostained for IBA1 (ionized calcium-binding adapter molecule 1, magenta), OTX2 (orthodenticle homeobox 2, yellow), the nuclei-dye Hoechst (blue) and brightfield image at week 18. Scale bar: 100 µm. **C**, Experimental schematic of adding branched cells from the supernatant of BMP4-guided protocol to 3D-retinal organoids without cystic compartment generated with unguided protocol (condition 2). **D**, Bar chart of number of IBA1$^+$-cells integrating into the structure and standard error at week 17 after 10 days of adding IBA1$^+$-cells. Each dot represents a 3D-retinal organoid. Green, condition 1 related to **A**-**B**. Magenta, condition 2 related to **C**, **E**. **E**, Whole mount 3D-retinal organoid without cystic compartment immunostaining like in **B**. Wilcox test p-value = 0.0436. $^*p < 0.05$.

Figure S8 (related to Figures 1-3, S3, S5)

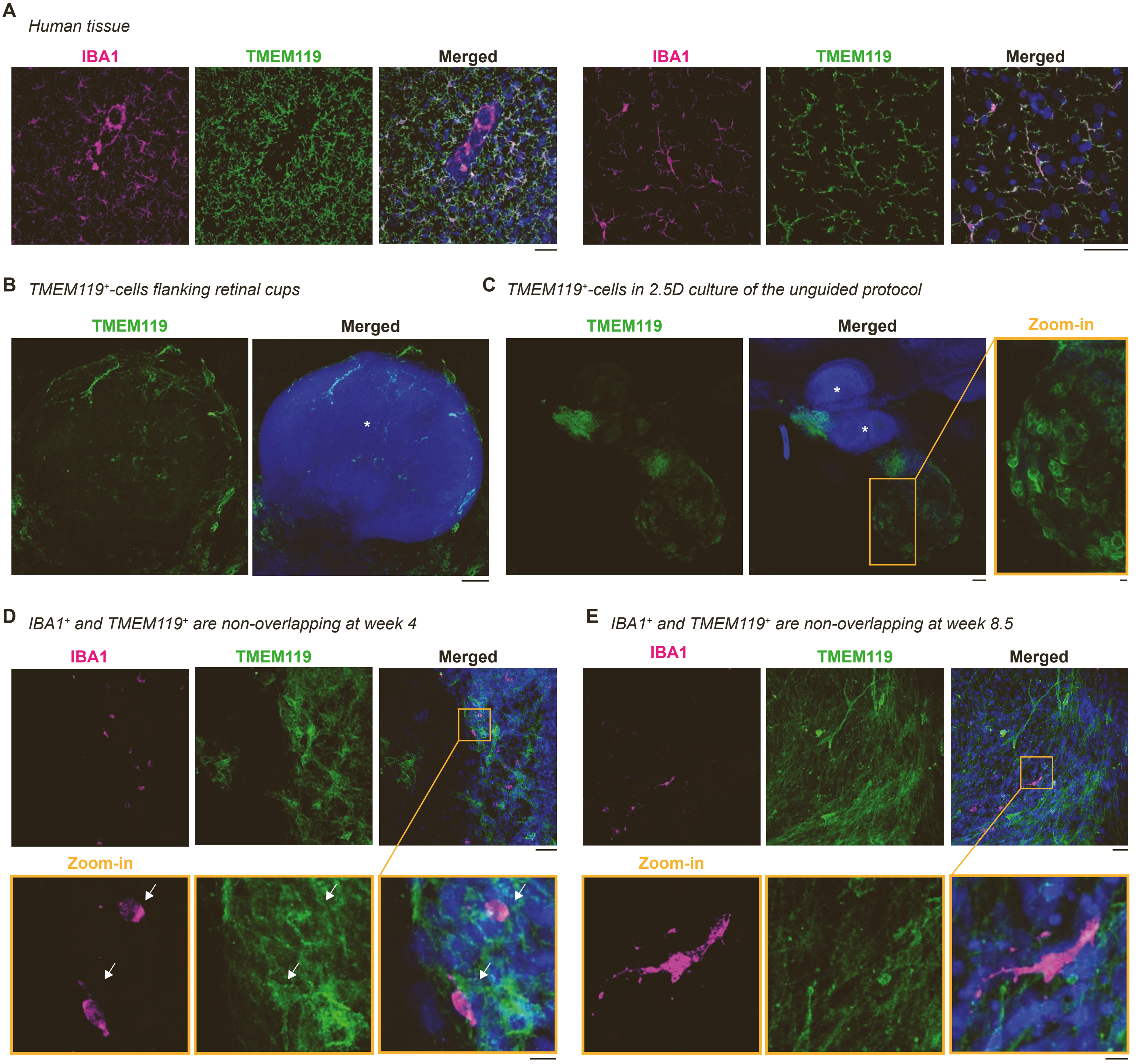

Figure S8. TMEM119 does not overlap with IBA1$^+$-cells (related to Figures 1-3, S3, S5).

Immunostaining for TMEM119 (Transmembrane Protein 119, green), IBA1 (ionized calcium-binding adapter molecule 1, magenta) and the nuclei dye Hoechst (blue). **A**, Two examples of vibratome sections of adult human temporal lobe. Left example with a blood vessel in a center that does not express TMEM119. Scale bar: 50 µm. **B-E**, 2.5D culture of unguided protocol from SC102A at week 4.5 (**B**-**D**) and week 8.5 (**E**). White *, cell-dense area. Scale bar: 50 µm. Orange frame: zoom-in with scale bar: 10 µm. Arrows: IBA1$^+$-cell embedded within TMEM119 side but not overlapping.
